# PrivEdge: a hybrid split–federated learning framework for real-time electricity theft detection on edge nodes

**DOI:** 10.1038/s41598-026-39064-8

**Published:** 2026-03-21

**Authors:** Ahmed Ramadan, Marwa A. Shouman, Gamal Attiya, A. S. ZeinEl Din, Elhossiny Ibrahim

**Affiliations:** 1 Department of Electrical Engineering, Faculty of Engineering, Menoufia University, Shebin Elkom, Egypt; 2 Computer Science and Engineering Department, Faculty of Electronic Engineering, Menoufia University, Menoufia, Egypt

**Keywords:** Electricity theft detection, Split–federated learning, Edge artificial intelligence (Edge AI), Privacy-preserving smart grids, Energy science and technology, Engineering, Mathematics and computing

## Abstract

Electricity theft is one of the primary contributors of non-technical losses in contemporary power grids, and traditional centralized methods of detection are limited in scale, feature a large communication cost, and create privacy issues. The presented paper introduces PrivEdge, a deployment-friendly hybrid Split–Federated Learning (SL–FL) system to detect real-time electricity theft on resource-constrained edge devices. PrivatEdge uses a Raspberry Pi 4-based smart meter gateway to do localized preprocessing with the Raspberry Pi 4 smart meter gateway and run a lightweight LSTM-based FrontNet; server-side functionality does more in-depth model inference, collaborative coordination, ensemble stacking, and score-level fusion. Split Learning allows conveying small intermediate activations as opposed to raw consumption data, which significantly lowers communication costs and minimizes privacy loss. Federated Learning supports distributed learning between highly non-IID clients who are geographically well-spread. Privacy maintenance is realized by secure aggregation and Laplace differential privacy, where ε = 3 is used as a uniform operation compromise due to practical consideration. As a high-security deployment mode, homomorphic encryption is supported. Extensive experiments on the SGCC smart meter data with IID and non-IID conditions reveal that PrivEdge would perform better in terms of detection accuracy and F1-score than both centralized and FL-only or SL-only baseline frameworks, especially in non-IID conditions. The software-level assessment using Raspberry Pi 4 hardware establishes a low inference time, consistent resource consumption, and endurance at that rate using sustained load. Ablation experiments also confirm the importance of localized preprocessing, time expression, ensemble-based aggregation of data, and their privacy-conscious learning. In general, PrivEdge helps in closing the gap between hybrid concepts of SL–FL learning and the practical needs of deployment in privacy-aware electricity theft detection at the network edge.

## Introduction

Electricity theft is one of the most acute issues of the current power utilities because it directly impacts the revenue streams and endangers the sustainability and reliability of the grid. The international estimates are that non-technical losses, which are mostly made up of electricity theft, can amount up to USD 100 billion every year on the planet^1^, and in certain areas can be as much as close to 40 percent of the electricity that is distributed^2^. In addition to the loss of money, such practices add a strain to the operation of the power systems and lower the service quality among legitimate consumers.

The implementation of smart grids and the use of advanced metering infrastructure (AMI) have made possible the acquisition of high-resolution electricity consumption data, which opens a new possibility in data-driven electricity theft detection. Nonetheless, the risk of privacy of users, communication delay, scaling, and computation overhead among utility providers presents a grave concern when it comes to central processing of fine-grained consumption traces^3^. These issues are even exacerbated by tough data protection laws, including the General Data Protection Regulation (GDPR) which place hard demands on collecting, transmitting and processing consumer data^4^.

Although there are elaborate smart meter data available, the majority of current electricity theft detection systems still utilize centralized learning systems. These solutions necessitate constant communication of raw or bitingly processed information to central servers with the resultant high communication overhead, slow detection, and single point of failure. Furthermore, massive, centralized networks find it hard to work effectively in the conditions of heterogeneous and unreliable wide-area networks and do not always comply with current privacy and regulatory requirements.

In order to work around such constraints there is growing interest in distributed and privacy-sensitive learning paradigms. FL allows distributed model training with collaborative training on distributed clients that do not have access to raw consumption data and has shown encouraging results in electricity theft identification tasks, including federated models built on ConvGRU training on identity issues with high detection error and privacy protection^5^.

However, FL based solutions continue to have high computational requirements on edge machines, are sensitive to non-independent and identically distributed (non-IID) data and incur high communication expenses as they require frequent model updates.

Split Learning (SL) has become an analogous paradigm, which divides the training of neural networks between edge devices and a server, and only intermediate activations are sent as opposed to the complete training model or raw measurements. The method also lowers the computational load of resource-constrained devices and improves privacy protection^6^, and multiple studies have found the method to be effective in IOT and edge computing environments^7^.

More recently, hybrid schemes between FL and SL have been suggested to enhance even more efficiency and privacy of distributed systems, including edge-assisted Split–Federated learning models^7^.

Nonetheless, a detailed hybrid Split–Federated Learning algorithm that is explicitly trained to detect a real-time electricity theft and that is verified with real-life smart grid data, i.e., the SGCC dataset^8^, has not been explored fully. The literature mainly concentrates on centralized models, single FL or SL models or hybrid models, which have been tested in simulated or cloud-centric environments but there is no information on how they can be realized in reality under realistic edge computing conditions.

To fill this gap, this paper will suggest PrivEdge, a Split–Federated Learning hybrid model, specifically designed to identify electricity theft on resource-constrained edge devices in real time. Contrary to the previous work, PrivEdge is developed, deployed, and experimentally tested against real-world edge hardware (Raspberry Pi 4) with real-world smart meter data. The suggested architecture will consider the detection accuracy, communication efficiency, privacy preservation, and the resilience to non-IID distributions in data simultaneously and provide a viable and scalable solution based on the modern-day smart grid and data protection needs.

The main contributors of this work are listed as follows:

- We present deployment-oriented hybrid Split–Federated Learning in real-time electricity theft detection carefully tailored on realistic edge computing setup other than idealized or cloud-centric setup.
- Develop, validate and implement the proposed framework experimentally on resource constrained Raspberry Pi 4 edge devices in a realistic setting using real-life smart meter data from SGCC dataset, demonstrating feasibility on both IID and non IID data distribution.
- We take temporal deep learning and ensemble-based decision fusion in privacy-aware way, by combining localized preprocessing and split inference, server-side aggregation and stacking, in order to improve the robustness against diverse theft pattern.
- In addition to detection performance, this work explicitly evaluates system-level feasibility, including communication efficiency, edge resource usage, scalability, and privacy–utility trade-offs.

PrivEdge does not present a new SL–FL optimization algorithm, but the systematic, deployment-oriented, and security-conscience implementation of hybrid SL–FL in the electricity theft-detection application and systematic empirical validation of privacy and poisoning attacks under natural edge-conditions.

## Related work

The electricity theft detection research has progressed considerably as the use of smart grids and the focus on privacy-conscious and edge-deployable learning algorithms has increased. The current literature can be roughly divided into four key directions, namely centralized deep learning, Federated Learning (FL)-based, Split Learning (SL)-based, and hybrid models consisting of both FL and SL.

### Deep learning models (DLM) that are centralized

Conventional electricity theft detection methods are mostly based on centralized deep learning models that include Long Short-Term Memory (LSTM) and CNN-LSTM models that have shown considerable potential in learning time and space consumption trends through smart meter data.

An example is the centralized CNN-based model that was suggested in^9^ and had high detection accuracy due to centrally aggregated smart meter readings.

Although they are effective, these centralized models necessitate complete access to raw data of user consumption which has serious privacy implications, is costly to communicate and has minimal scalability in large scale smart grid systems.

### Federated learning-based adventures

As an alternative to sharing raw consumption data, Federated Learning has been proposed as a decentralized model-training protocol that can achieve collaborative model training. The^5^, federated hybrid deep learning framework was suggested to enhance the detection of electricity theft in an Industry 5.0 setting, which proved better privacy preservation and competitive results. Similarly,^10^ introduced a deep anomaly detection scheme that is FL-based and can detect zero-day electricity theft cyber-attacks without data privacy breach. In order to overcome the issue of data heterogeneity and class imbalance, a heterogeneous FL model that incorporates CNNLSTM architectures and CKKS homomorphic encryption was proposed in^11^, with better detection performance, and without the loss of privacy.

Though FL-based techniques are useful in minimizing privacy risks, they are commonly characterized by high computational costs on edge devices, slow convergence when using non-IID data distributions, and high communication costs as a result of frequent model parameter updates.

### Split learning-based methodologies

Split Learning provides another paradigm of distributed learning: the code of the neural network is split into halves, with one half running the network in clients and the central server, where it is physically isolated, is exposed to as little sensitive data as possible. In^12^ the use of SL based scheme to detect electricity theft with demand response technique was suggested which proved to be more efficient with less privacy loss. Moreover,^13^ proposed a Transformer-based SL model improved with Generative Adversarial Networks (GANs) and Elliptic Curve Diffie Hellman (ECDH) cryptography to further improve privacy protection. Although SL-based systems mitigate edge-side computation and privacy issues, coordination and scaling across multiple distributed clients is still a challenge to implement in large-scale smart grid systems.

### Federated-split learning hybrid frameworks

Hybrid to Federated Learning and Split Learning could be distributed by mixing the efficiency of communication, the computation load and privacy protection in both paradigms. In^7^, an Edge-assisted U-shaped Split–Federated Learning (EUSFL) system was presented, and it achieved better privacy, latency and energy efficiency in IOT systems. Likewise, FL and U-shaped SL were employed together with the FUSE framework in^14^ to obtain the correct and privacy-aware electricity theft detection. Although with good performances, the majority of hybrid FL-SL is tested in the cloud or virtual IOTs, and there are few tests in the real edge computing conditions.

A summary of relevant studies in the centralized and FL-based, SL-based and hybrid-learning paradigms with their respective methodology, privacy mechanism, detection performance and communication cost are summarized in Table 1.

**Table 1:** Summary of related work.

Category	Author & Year	Methodology	Data type	Privacy protection	Detection performance	Communication cost	Ref.
Centralized DL	Haque et al., 2022	Deep CNN for electricity theft detection	Centrally aggregated data	Low	High with sufficient data	High	^9^
FL	Zafar et al., 2023	Hybrid Deep Learning assisted by FL in Industry 5.0	Distributed	High	High	Medium	^5^
FL	Alshehri et al., 2024	Deep Anomaly Detection using FL for Zero-Day attack detection	Distributed	High	High	Medium	^10^
FL	Wang et al., 2025	HeteroFL (CNN-LSTM + CKKS Encryption) for handling data imbalance	Distributed	High	High	Medium	^11^
SL	Alromih et al., 2023	SL with Demand-Response for energy theft detection	Partially distributed	Very high	High	Low–Medium	^12^
SL	Yang et al., 2024	Transformer-SL + GAN + ECDH for secure energy theft detection	Partially distributed	Very high	High	Low–Medium	^13^
Hybrid FL—SL	Tang et al., 2023	Edge-assisted U-Shaped Split–Federated Learning for IoT data processing	Fully distributed with model splitting	Very high	Very high	Low	^7^
Hybrid FL—SL	Li et al., 2024	FUSE: Hybrid FL + U-Shaped SL framework for electricity theft detection	Fully distributed with model splitting	Very high	Very high	Low	^14^

Current studies, as the literature review shows, have been actively pursuing the field of Federated Learning, Split Learning and hybrid FL-SL, as ways of detecting electricity theft. Nevertheless, most of the currently available solutions are either centralized or cloud-centric, or are based on idealized conditions of the edges, or not experimentally tested on real world edge hardware. Further, the collective analysis of the risks of activation leakage, effectiveness of communication, and solidarity of decisions in an ensemble-based in a fully deployable edge structure has not been examined comprehensively. Such restrictions lead to the creation of a multifaceted, privacy-conscious, and edge-native solution that is the direct inspiration of the suggested PrivEdge framework.

Purely on-device lightweight models offer reduced complexity but typically suffer from limited generalization, lack of collaborative learning, and reduced robustness to non-IID consumption patterns, motivating the need for hybrid SL–FL approaches despite their architectural complexity.

### Positioning of PrivEdge relative to existing hybrid SL–FL frameworks

Most recent hybrid Split–Federated Learning architectures, including FUSE and EUSFL, have shown that distributed intelligence can be achieved by using a combination of split learning and federated learning. Nevertheless, the focus of these works is mainly on the accuracy of the algorithms under simulated or clouded conditions and convergence, and little focus is on deployability requirements to resource-constrained edge hardware. PrivEdge on the contrary is clearly a deployment-focused system. It adds value not by suggesting a novel variable of hybrid learning, but by offering a confirmation analysis of practical real-time electricity theft detection under realistic edge conditions in a systematic and hardware validated methodology. This encompasses long-run execution (24 h), measured resource use (CPU, memory, energy, and thermal behavior) which is robust to controlled network impairments, multi-meter-based inference by batches as well as direct analysis of privacy-latency-utility trade-off. Table 2 gives the main differences between PrivEdge and current hybrid SL–FL frameworks, their focus on deployment and system-level validation.

**Table 2:** Positioning of PrivEdge relative to existing hybrid SL–FL frameworks.

Aspect	FUSE / EUSFL	PrivEdge
Hybrid SL–FL concept	✓	✓
Real edge hardware (Raspberry Pi)	✗	✓
Long-duration execution	✗	✓ (24 h)
System-level overhead analysis	Limited	Comprehensive
Network impairment evaluation	✗	✓
Batch-based multi-meter inference	✗	✓
Privacy–utility–latency quantification	✗	✓

PrivEdge, therefore, does not purport to innovate the hybrid SL–FL learning paradigm as such,

but instead of offering a deployment-oriented, system-verified implementation of such paradigms.

under real-world edge constraints in electricity theft detection. Therefore, PrivEdge’s novelty lies in its system-level validation under real deployment constraints rather than proposing a new learning algorithm.

## Proposed methodology

### System overview

**Fig. 1:**
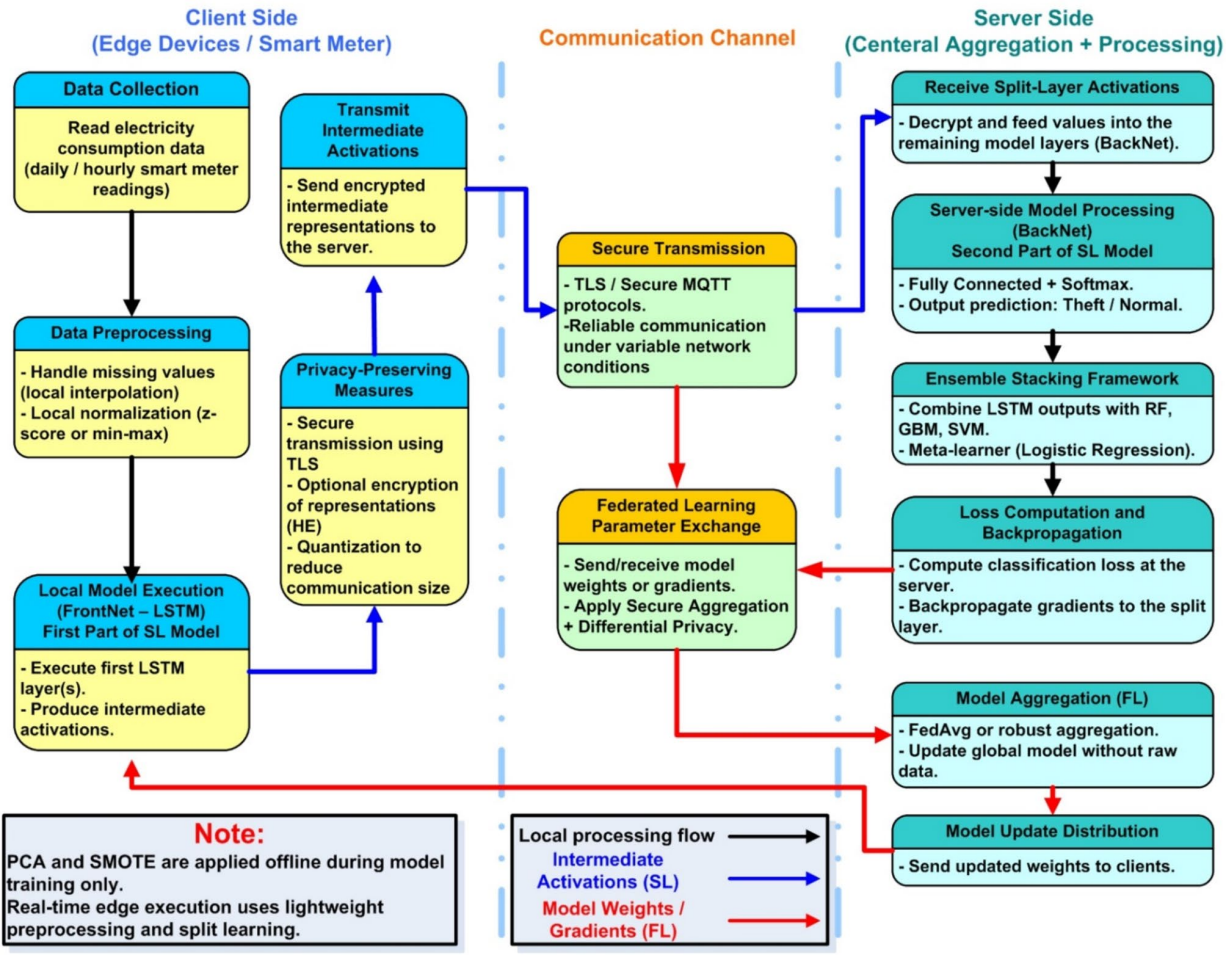
Illustrates the overall training workflow of the proposed PrivEdge framework.

Figure 1 is the general structure of the proposed PrivEdge framework, which follows a basic Split–Federated Learning (SL–FL) paradigm of real-time electricity theft detection under realistic edge computing requirements. In comparison to cloud-centric, PrivEdge is designed specifically to run on resource limited edge devices. Under this architecture, every smart meter gateway, which is a Raspberry Pi 4 Model B, will run local data preprocessing and partial model execution and any higher-level learning coordination, aggregation, and decision fusion will be performed by a central server.

As illustrated in Fig. 1, PrivEdge is a systematic end-to-end workflow pattern in accordance with the real-world smart grid workings. Smart meter gateways at the edge layer simultaneously obtain and accept electricity consumption information in live Advanced Metering Infrastructure (AMI) streams, or historical data like SGCC. Raw consumption measurements are first processed locally to deal with missing values, extract information features, decrease dimensionality, and alleviate class imbalance and then fed to the learning pipeline. This architecture means that raw consumption traces of the high-resolution are not lost on the cloud servers which immediately provides privacy protection needs and regulatory compliance.

PrivEdge balances the privacy protection, computational efficiency, and communication overhead with the help of Split Learning, which splits the deep temporal model into a lightweight FrontNet, which runs on the edge device, and a computationally intensive Back-Net, which runs on the server. The server receives only intermediate activations that are caused at the split layer. This method uses much less bandwidth as compared to centralized learning, and the information is also less leaked than complete sharing of model parameters. The splitting point is chosen critically to reduce the effect of computation on edge computers but at the same time to keep the representation transmitted small and naturally fit within edge computer limitations. Early placement of the split would greatly multiply the dimensionality of the activations transmitted, suffering additional bandwidth usage and possible information leakage. In contrast, more profound divisions would be computationally and memory-intensive to resource constrained edge machines like the Raspberry Pi 4. The chosen split set up thus offers a sensible trade-off in that lightweight edge execution is achieved and the smaller and task-relevant representations of the data are relayed to the server.

Besides split learning, Federated Learning is also incorporated to organize collaborative training among two or more edge devices. The updates to the model provided by the participating clients are regularly summed up at the server based on the standard federated aggregation schemes, including FedAvg. This hybrid coordination mechanism allows PrivEdge to scale to geographically distributed smart meters, and yet to be resilient to non-IID data distributions (heterogeneous data) and network conditions, and yet to reveal no local consumption data.

PrivEdge has an ensemble-based decision module at the server layer, which integrates a variety of classifiers, such as the Random Forest and Gradient Boosting, and Support Vector Machine models. These classifiers are working on high level features that are obtained by the Back-Net and are combined by the use of stacking strategy in order to increase the detection resilience to various and changing electricity theft modes. Decisions on final anomaly detection and alert generation are made centrally, but optional pre-screening at the edge is used to minimize latency on the obvious cases.

In general, the proposed architecture clearly responds to the practical limitations that were discovered in previous research by collectively managing the edge resource constraints, communication efficiency, privacy threat, and decision robustness in the same deployable architecture. The design decisions shown in Fig. 1 can be used to make PrivEdge adaptive to smart grid applications in the real world, where devices are heterogeneous, data is not ideally distributed, and network parameters are dynamic.

The division point between the FrontNet and Back-Net is chosen to optimize three antagonistic goals, namely, reducing edge-side computation, minimizing communication overhead, and minimizing information leakage of intermediate activations.

Low-level representation of temporal consumption patterns are captured by early layers, which are also lightweight enough to run on Raspberry Pi 4 hardware, and deep layers are used to perform higher-level abstraction and run on the server.

This option guarantees the feasibility of deployment on resource-constrained devices in the edge and has enough representational ability to detect theft.

### Data preprocessing

This preprocessing pipeline has been designed to balance the two main aspects of robustness and efficiency of computations. Computationally intensive operations (e.g. PCA and SMOTE) are implemented during offline training and only lightweight computations such as handling missing values and normalizing features are implemented during real-time edge inference for maintaining low-latency performance on resource constrained devices. Electricity theft detection datasets often exhibit high dimensionality, noise, and class imbalance. In real-time edge deployment, local processing (lightweight) tasks (e.g. missing values and feature normalization) are only done in the smart meter or edge gateway. This isolation is to make sure that computationally intensive steps do not interfere with real time performance on resource constrained machines. In order to reduce the possible bias caused by synthetic oversampling, SMOTE is implemented in a conservative way following dimensionality reduction, and the ratios between the oversampling are adjusted in such a way that the genuine consumption pattern is not distorted. Moreover, the federated training process allows one to be continuously adapted to changing theft patterns, to minimize over fitting to synthetic samples and to increase the ability to be robust to non-stationary consumption patterns. To address these challenges, the following pipeline is executed locally on each Raspberry Pi 4 client^15^:

#### Missing data handling

The smart meters have usual data gaps due to the malfunctions of hardware and transmission errors in data sampling. Inadequate recording of data poses significant problems to the analysts as they require powerful procedures or methods of missing data imputation^16–18^.

To overcome such problems, a temporal nearest-neighbor interpolation was achieved with a mathematical definition (Eq. 1) of the study.

1 $$\hat{x}_{i} = \left\{ {\begin{array}{*{20}l} {x_{i} } \hfill & {if\:x_{i} \:is\:observed} \hfill \\ {\frac{{x_{i - 1} + x_{i + 1} }}{2}} \hfill & {if\:x_{i - 1} \ne \:NaN \wedge \:x_{i + 1} \ne \:NaN} \hfill \\ {x_{i - 1} } \hfill & {if\:x_{i + 1} = NaN} \hfill \\ {x_{i + 1\:} } \hfill & {if\:x_{i - 1} = NaN} \hfill \\ \end{array} } \right.$$

Let ${{x}}_{{i}}$ denote the electricity consumption measurement at time index $i$. Missing observations are denoted as ${NaN}$. When a missing value occurs, local temporal interpolation is applied using the nearest available neighbors ​${{x}}_{{i}-1}$ and ${{x}}_{{i}+1}$​, as formalized in Eq. (1). The approach relies on the immediate measurements before and after (${{x}}_{{i}-1}$ and ${{x}}_{{i}+1}$) to compute the missing values by conducting an arithmetic average in case there are fleet gaps in the missing data. This temporal local interpolation approach is resistant to short-term missing values without applying any bias to the results on a global scale since local interpolation only uses local temporal neighbors.

#### Feature extraction and scaling

Time-series are windowed (e.g., 24-h segments) and transformed into engineered features: daily total usage, peak-hour load, off-peak ratio, and consumption change rates^19^. Feature scaling via MinMaxScaler is performed locally to avoid leakage of scaling parameters^20^. The study employed Eq. (2) for carrying out scaling procedures.

2 $$F\left({x}_{i}\right)=\frac{{x}_{i}-Min (X)}{Max \left(X\right)- Min (X)}$$

where, is ${Min }({X})$ is the minimum value in X and ${Max }\left({X}\right)$ is the maximum value in X.

The feature engineering and scaling are done locally at every client to ensure that the global statistics are not leaked and that non-IID consumption patterns among the various households are accommodated.

#### Dimensionality reduction (PCA)

Principal Component Analysis (PCA) is used as a preprocessing tool in the first place to decrease the number of features and stabilize further learning with limited resources. PCA has not been applied as a single anomaly detector in PrivEdge, but it is a compact representation mechanism that retains the major consumption patterns but removes noise and redundant correlations. The PCA technique removes massive high-dimensional numerical data by developing lowered-dimensional forms. The resulting anomaly score calculated in this way is compared with some predefined threshold in order to indicate the presence of anomalies^21–24^.

The electricity consumption data are presented as a matrix X the dimensions of *m* × *n* which m and n represent counts of observations, and variable numbers respectively. The key first principal component is the outcome of:

3 $$p1= {w}_{1}^{T} X= {w}_{11 }{x}_{1}+ {w}_{12 }{x}_{2}+\dots + {w}_{1n}{x}_{n}= \sum_{j=1}^{n}{w}_{1j}{x}_{j}$$

In Eq. (3) the coefficients ${{w}}_{2}={( {{w}}_{21}, {{w}}_{22},\dots , {{w}}_{2{n}})}^{{T}}$ that maximize the variance $var \left(p2\right)= {w}_{2}^{T} {\sum }_{x}w2$ are constrained by ${\left|w1\right|}^{2}= {\sum }_{j=1}^{n}{w}_{1j}^{2}=1$. That is because the first principal component has the highest variance in the data. On the same note, the second principal component is described as:

4 $$p2= {w}_{2}^{T} X= {w}_{21 }{x}_{1}+ {w}_{22 }{x}_{2}+\dots + {w}_{2n}{x}_{n}= \sum_{j=1}^{n}{w}_{2j}{x}_{j}$$

To maximize the variance in Eq. (4), we set the coefficients ${w}_{2}={( {w}_{21}, {w}_{22},\dots , {w}_{2n})}^{T}$ so as to maximize the quadratic form $var \left(p2\right)= {w}_{2}^{T} {\sum }_{x}w2$ subject to the constraint ${\left|w2\right|}^{2}= {\sum }_{j=1}^{n}{w}_{2j}^{2}=1$ with the further requirement $\left[{w}_{2} {w}_{1}=0\right]$; this makes orthogonal to the first principal component. Consequently, the second principal component accounts for the second biggest variation of the data.

Then, the principal components are defined in $n$ decreasing order of variance. The vectors ${w}_{1}, {w}_{2},\dots , {w}_{n}$ of coefficients are called the loadings and the respective main components are denoted ${p}_{1}, {p}_{2},\dots , {p}_{n}$. We choose to reconstruct the data with these principal components, since most of the variance in the dataset would be captured there and the first few components.

PCA is traditionally sensitive to noise, however, when applied to localized interpolation, window-based feature aggregation, and normalization, it becomes much more insensitive to sporadic errors in measurement. Furthermore, PCA is solely used as a dimensionality reduction mechanism but not as an anomaly detector that maintains discriminative information to be used at later learning phases and enhances computational efficiency and communication performance.

#### Class balancing—synthetic minority oversampling technique (SMOTE)

Post-PCA is used to create synthetic minimal-class (theft) examples that can increase the classifier sensitivity to aberrant patterns. K-neighbors and sampling ratios are hyper parameters that are tuned to constraints of deployment^25^.

The SMOTE works based on a linear interpolation which connects minority class specimens to their nearest neighbors and generates synthetic events. It takes the following steps:

- Consider a particular sample within the set of minority classes, ${{x}}_{{i}}$ and find the Euclidean distance between that sample, ${{x}}_{{i}}$ and the rest of the samples within the whole set. Find k of its neighbors, which will be referred to as ${y}_{j}$ (j = 1, 2, …, k).
- The sampling rate is decided upon depending on ratio of data imbalance to determine the oversampling factor. In a sample ${{x}}_{{i}}$, n neighbors are randomly picked among the k-nearest neighbors, and the new synthetic samples are created in the following way:
  5 $${x}_{new}={x}_{i}+rand(0,1)\times ({y}_{j}-{x}_{i})$$

**Fig. 2:**
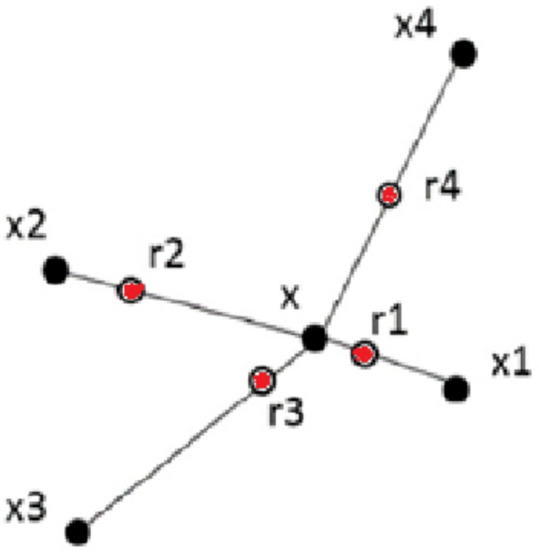
New data synthesized by SMOTE.

Here ${x}_{j}$=1, 2, …, n and Rand (0,1) are random numbers bounded between 0 and 1. Figure 2 shows new data synthesized by SMOTE.

To preclude the high-level synthetic bias, SMOTE is provided in a conservative way in terms of dimensionality reduction, that is, the feature space is smaller and less polluted. Oversampling and neighborhood ratios are also optimized to fit the actual consumption pattern of theft to reduce the chances of introducing bias in the actual consumption patterns.

In the real-world applications of smart grids meter readings are typically corrupted with noise, non-sample and non-uniform reporting intervals due to device malfunction or communication failure. The preprocessing pipeline is therefore specifically designed to be lightweight and robust in that it can be deployed in such a scenario by leveraging localized interpolation, window-based feature aggregation and dimensionality reduction in order to absorb the sporadic noise effects. This is compounded by time modeling and ensemble decision schemes which we use subsequent to eliminate the impact of preprocessing imperfection.

Regarding the control of imbalance in classes, the SMOTE is applied after choosing the features and reducing their dimensions in order to minimize the distortion of the original patterns of consumption. The oversampling parameters are not overly aggressive in a way that they result in an excessive amount of synthetic bias, and the federated training process allows the model to continuously adjust based on the new real consumption patterns. This design must ensure that it becomes sensitive to infrequent thefts, and also robust to non-portable consumption behavior.

### Model architecture and base learners

The system is actually based on a heterogeneous ensemble to make use of temporal and static consumption attributes:

#### LSTM (FrontNet)

Long Short-Term Memory (LSTM) architecture is chosen because it is shown to be very effective in long-range temporal dependencies of electricity consumption time series where stealing patterns are often gradual changes and not spikes. The split point of Split Learning configuration is created in such a way that it becomes possible to implement a balance between parts of the computational performance, preservation of privacy, and overhead of communication provisions, where the balance line is introduced after the first recurrent LSTM layer. The option is such that the majority of uncooked time data is stored on the client, and the sent intermediate activations are small and less informative in privacy terms. This split design is empirically dictated to offer a desirable tradeoff between edge-side resource constraints and model expressiveness thereby being easily deployed to resource-limited devices like Raspberry Pi 4 Model B.

**Fig. 3:**
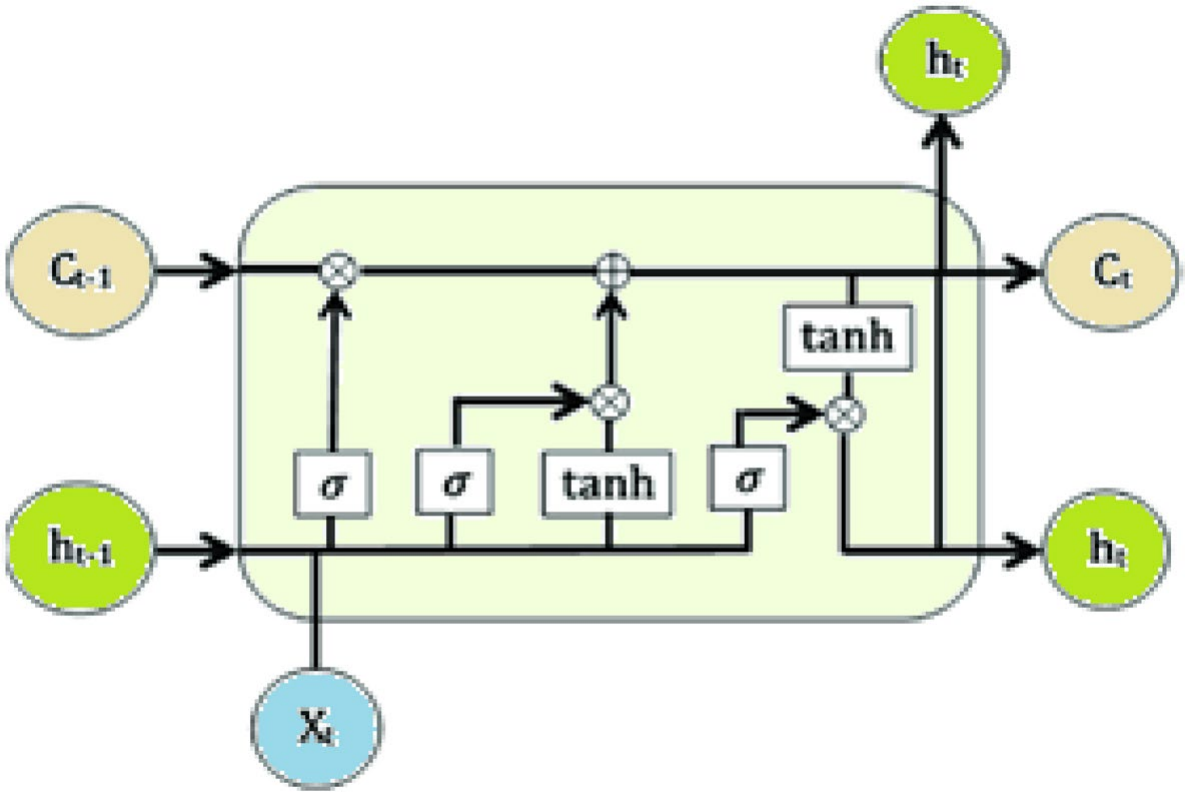
The LSTM architecture.

Lightweight, 1–2 layers with ~ 64 hidden units, deployed on the client to capture temporal dependencies. LSTM networks comprise a type of Recurrent Neural Networks (RNN) that has overcome the vanishing gradient problem. According to^26–30^. LSTMs exhibit long-term contextual relations because their memory cells have input, output and forget gates as shown in Fig. 3:

Formula (6) is used to obtain the formation of the forget gate.

6 $${f}_{t}= \sigma ({W}_{f}.\left[{h}_{t-1}, {x}_{t}\right]+ {b}_{f})$$

A forget gate is used and this applies both sigmoid and with $tanh$ to regulate the information added to the cells state. Each of the two functions takes as input the previous hidden state (${h}_{t-1}$) and the current input (${x}_{t}$). Importance of information is determined by using sigmoid function whereas biasing or controlling the network is observed by using $\mathrm{tanh}$ function where a restriction on the value in the network occurs between -1 and + 1. The final step multiplies the two outputs of functional outputs.

7 $${i}_{t}= \sigma \left({W}_{i}.\left[{h}_{t-1}, {x}_{t}\right]+ {b}_{i}\right)$$

8 $${\widetilde{C}}_{t}= \mathrm{tanh}({W}_{C}.\left[{h}_{t-1}, {x}_{t}\right]+ {b}_{C})$$

The outputs of the forget gate and the input gate are used in updating the cell state. It is performed with the help of pointwise multiplication of the current cell state and the output of the forget gate.

When ${f}_{t}$ = 0, the outcome of the multiplication will be 0, i.e.: the former information will be totally neglected. However, when ${f}_{t}$ = 1 then the information is maintained. The last process is to update the cell state by a pointwise addition.

9 $${C}_{t}= {f}_{t}\times {C}_{t-1}+ {i}_{t}\times {\widetilde{C}}_{t}$$

The output gate determines which information is to be taken to final output and as the next hidden state ${h}_{t}$ in the next and final step of processing. The sigmoid function involves procession of two inputs, previous hidden state ${h}_{t-1}$ and the current input ${x}_{t}$ along with $\mathrm{tanh}$ processing current cell state ${C}_{t}$. One of these functions is multiplied by another, and thus the information retained by the hidden layer is decided.

10 $${S}_{t}= \sigma ({W}_{o}.\left[{h}_{t-1}, {x}_{t}\right]+ {b}_{o})$$

11 $${h}_{t}= {S}_{t}\times \mathrm{tanh}({C}_{t})$$

#### Random forest & gradient boosting:

Tree-based learners that identify complex, non-linear feature relationships. Random Forests constitute an ensemble learning system in the sense of being constituted by several decision trees. The Decision Tree models, coupled with the ensemble methods work by obtaining historical data on load to determine artificial electricity theft activities against normal electricity usages^22^. According to^31^, gradient boosting is a common algorithm selection with regard to handling regression tasks. Due to the weight on avoiding overfitting effects, gradient boost algorithms are more prone to introducing stricter constraints of model development as compared to random forest.

#### Support vector machine (SVM):

SVM is a supervised machine learning algorithm^32^ that is devoted towards differentiating genuine and counterfeit electricity consumption patterns. The points that the support vectors occupy assist in drawing a line of distinction between a legitimate pattern of using electricity and the present use of electricity as an act of fraud to enhance accuracy of detecting electricity theft.

#### Stacking meta-learner:

A meta-learner was trained by utilizing outputs of the base learners using cross-validation cycles. The logistic regression will be used as a meta-learner in this paper as it has the ability to acquire predictions of linear combination of different models^33^. The RF, the GB and the SVM output was fused at the meta-level to create a new training dataset over each fold of the validation set.

The combined dataset only had the number of rows equal to the initial dataset with the extra features being the output of the predictions of the base learners. The labels that still had true classes were involved in the procedure to ensure that the meta-learner is trained correctly in accordance to^34^. This reformulated dataset was passed to the trained Logistic Regression model in order to obtain appropriate values of the weights that would join the base learner forecasts. Stacking ensemble methods are useful in binary fraud classification since it allows the models to capitalize on individual strengths and overcome their disadvantages to produce better results than that of the base learners alone. Implementation occurs as follows^35,36^:

- The data must be divided into two: testing data (20 percent) and training data (80 percent).
- All the base models which include RF and a combination of GB and SVM incorporate a cross-validation method of training.
- Cross-validation predictions of base models will be stacked horizontally and form a single new dataset.
- The meta-learner is trained using labels to compile the training data bestowed with real stacked prediction.
- Upon using the stacking procedure on test data, trained meta-learner would receive its input in terms of generated features.
- The logistic regression meta-learner must be trained with the training set which has been just made.
- The meta-learner will be evaluated by accuracy measurements in conjunction with precision, recall and F1-score success measures on test set.

The ensemble stacking framework is implemented only at the server side where there are adequate computational resources to aggregate the temporal and non-temporal learners and make it more resilient to various electricity thefts.

Whereas the stacking meta-learner is now implemented at the server, the ensemble architecture is scalable to a distributed or hierarchical implementation across multiple edge gateways and can thus prevent a single point of failure.

### Hybrid FL and SL deployment strategy

This subsection provides an operational description of the proposed workflow to clarify the interaction between Split Learning and Federated Learning components, rather than introducing additional architectural modules. These steps outline logical operation flow of PrivEdge and do not reflect autonomous system modules, though coordinated phases in harmony with the architecture as displayed in Fig. 1. Under this hybrid architecture, Split Learning has the job of dividing the neural network between edge and server using intermediate activations and Federated Learning manages to achieve global model optimization by adding together encrypted weights or gradients at a collection of clients. The proposed deployment uses asynchronous and loosely coupled coordination strategy to reduce the architectural complexity created by the joint use of Split Learning and Federated Learning. The clients are involved in the training rounds as opportunity based on the local resources, and the server summarizes the updates with partial participation schemes. The design minimizes the synchronization delays, eliminates straggler effects, and has greater robustness when the workloads of the edges are heterogeneous, which suits the large-scale and real-world applications better. The end-to-end workflow operates as follows:

#### Data acquisition

The first, mission critical step is data acquisition: individual Raspberry Pi 4 Model B (or smart-meter gateway) acquires the household smart-meter or AMI feed at the configurable interval (the house reads generally occur daily, but also at a 15-min and hourly rate depending on the deployment and SGCC representation). Raw and short-circuit timestamps should be locally buffered (to recover gracefully after transient link losses); it is encouraged that the Pi save rolling windows and only present processed features to downstream stages. The SGCC dataset (State Grid Corporation of China) is a commonly used realistic labelled dataset that can be used in electricity-theft research and consists of ~ 42 k customers over ~ 1,000 + days, with experiments/validations on SGCC easily transferrable to many other electricity-theft studies. In practice, Raspberry Pi 4 Model B can do on-device preprocessing and light inference with optimized runtimes (TensorFlow Lite / ONNX Runtime) and quantized models but requires thoughtful RAM/CPU budgeting, thread scheduling and optional accelerators (Edge TPU, NCS) for production machine deployments^37^.

#### Local preprocessing (missed data, feature extraction, scaling, PCA, SMOTE)

Unfiltered meter readings must be processed, filtered and converted to in-device in order not to transmit raw time-series that could be identified. Standard actions:

- Linear interpolation fill-in small gaps, drop or statistically imputed long gaps to prevent false positives).
- Construct windowed features (sum by day/hour, peak/average ratio, hourly delta, weekday/weekend indicator, season indicator) by fix-sized windows (24 h, 7d, 30d) so that we can shed payload and still have information on time progression
- Locally scale features using Standard Scaler or MinMaxScaler (can keep scaler parameters local to avoid distributions leaking across blocks). SMOTE is a commonly used method of minority oversampling; PCA is a long-established method to reduce the dimensions of data. Most of the electricity-theft related works based on the SGCC data employ PCA/SMOTE variants in the preprocessing reducing in order to enhance recall among the theft classes^24,25,38^.

#### Partial model execution (SL)

Split Learning Splitting a deep model into a FrontNet (client side) and a Back-Net (server side) is possible in Split Learning (SplitNN). For every forward pass, the client will compute up to the split layer, forward to the server, and the server will do a forward pass and compute the loss. The gradients are back propagated back to the split by the server; the server returns the gradient to the client, who then performs back propagation on the FrontNet weights. In temporal models (LSTM-front/back), the split can be typically placed just after the first recurrence layer or a light convolutional front so that resulting activations remain small (e.g. 32–128 floats per window) and most raw signal remains on device. Split Learning decreases client computation and memory over fully local training and can be used together with FL aggregation of FrontNet weights across clients when needed (SplitFed variants). Nevertheless, the choice of split points has to trade-off between the size of activations, the leakage of privacy (activations must leak little information in order not to reveal anything or too much information to become useless), and computational complexity. These trade-offs and viable choices of contact with the edge devices are documented by empirical and theoretical split-learning works^39,40^.

#### Privacy preservation

As much as Split Learning (SL) and Federated Learning (FL) do not share raw electricity consumption time series, one should mention that the same sensitive information may still be leaked through intermediate activations and gradients. Model inversion attacks and gradient-based reconstruction attacks previous studies have shown that adversaries could retrieve sensitive information about a model user by reconstructing the users’ privacy data based on shared parameters or activations.

To avoid these threats, several privacy preserving defenses may be used:

- Activation Quantization and Compression—These methods include 8-bit quantization, pruning and Top-k sparsification to drastically reduce bandwidth consumption and leave out fine-grained numerical information that may be used by the adversary. Quantization-Aware Training (QAT) allows the model to stay highly accurate even with that quantization^41^.
- Differential Privacy (DP)—By using Differentially Private Stochastic Gradient Descent (DP-SGD) or applying some controlled noise to the activations and the gradients, it is possible to obtain mathematically verifiable privacy guarantees. The privacy parameter 2 (epsilon) should be well-adjusted in order to strike a balance between the usefulness of the model and its privacy protection^42^.
- Homomorphic Encryption (HE)—With respect to high confidentiality, one can employ Cheon-Kim-Kim-Song (CKKS) homomorphic encryption to update and activations. This enables server to do aggregation without decryption. CKKS is more expensive and latent, though with support of real-valued numbers approximate arithmetic^43^.
- Secure Aggregation Protocols -They include protocols that guarantee the server receives aggregated updates of several clients without knowing details from individually raw updates. When augmented with Differential Privacy (DP) and targeted use of Homomorphic Encryption (HE), such strategy provides a good protection against leakage of information with bounded computational cost and communication overhead^44^.
- Privacy-utility trade-offs should be assessed alongside each other empirically (e.g. via reconstruction attack simulations) and theoretically (formal privacy bound).

#### Secure transmission

All activation/model-update traffic should be over authenticated and encrypted traffic. Transport Layer Security version 1.3 (TLS 1.3) as defined in RFC 8446 should be used to transport from the client to server^45^. In the case of Internet of Things (IOT) deployments the typical pattern is the Message Queuing Telemetry Transport version 5.0 (MQTT v5.0) over TLS^46^ or secure Hypertext Transfer Protocol Secure/Representational State Transfer (HTTPS/REST) with mutually authenticated TLS^47^. Latency spikes, packet loss or sporadic connectivity may occur in communication links between real grid smart grid environments. To overcome such conditions, the proposed system supports buffered transmission, update-retry mechanism and delayed aggregation without interrupting local inference.

When connectivity is lost, edge devices will keep executing part of the models and screen local anomalies whereas global model updates are coordinated when communications become available again. On devices in which the use of a Public Key Infrastructure (PKI) is not feasible, rely on certificate pinning or pre-shared keys^48^. Lock down the broker/server through features like mutual TLS, short lived certificates and replay protection^49^, enforce strong cipher suites, provide TLS session resumption to support low latency and check certificate expiry^50^. In a case of intermittent connectivity, design resettable transmission mechanisms with checks, integrity and end to end authentication^51^.

#### Server-side processing

After getting encrypted activations of edge clients, the server-side BackNet finishes the forward and backward propagation of the Split Learning (SL) model to allow high-level feature extraction without being provided raw consumption data^39^. The server then combines a variety of classifiers Random Forest (RF) and Support Vector Machine (SVM) and Gradient Boosting Machine (GBM) to process and identify consumption abnormalities based on the activations. RF and GBM are successful in capturing non-linear feature interactions, whereas SVM offers strong margin-based discrimination to binary theft detection^31,52^. The results of the prediction of the above-mentioned base learners are stacked in a stacking meta-learner (usually a lightweight LightGBM or a logistic regression) that trains to weight the models in the best way. The meta-ensemble approach provides a substantial boost in generalization and minimizes overfitting behavior in a wide range of consumption spends^53^. Once local inferences have been received, the server arranges federated aggregation by computing an update of a global model over client participants. This learning procedure, commonly executed by Federated Averaging (FedAvg) or its sturdier alternatives, can guarantee that single updates will be confidential and in the course of improving the shared model constantly^54^. Any communication between the edge and the server is done over authenticated and encrypted channels—either version 1.3 of Transport Layer Security (TLS 1.3) or version 5.0 of Message Queuing Telemetry Transport (MQTT v5.0) over TLS—to ensure the secrecy and integrity of relayed activations and gradient stabilization and mitigates overfitting at varying consumption patterns^45,55^.

#### Federated aggregation

After the transmission of the current communication round, the associated clients relay encrypted weight or gradient changes to the central server. The server then conducts a global aggregation in form of Federated Averaging (FedAvg) that calculates the weighted mean of the updates according to the sample size of individual clients, thus updating the shared model without crossing the boundaries of raw data^54^. In order to alleviate the impact of adversarial clients or model poisoning, robust aggregation algorithms, such as Krum or Trimmed Mean, are used to drop outliers in gradients and ensure Byzantine fault tolerance^56,57^. At set intervals, the server can access validation metrics or a select reference dataset in order to identify model drift and initiate a fall back in the event of need.

#### Model update distribution

The new model parameters are shared back to all participating clients after every global aggregation to update the model parameters. Every client substitutes its local model with global parameters and carries training in its local data. Federated Proximal Optimization (FedProx) and Federated Normalized Averaging (FedNova) methods may be used to deal with system heterogeneity as an alternative to the traditional Federated Averaging (FedAvg) algorithm. In FedProx, a proximal term is added to the local optimization problem, and this tends to reduce the difference between local model changes and the global model, thereby enhancing the convergence stability in non-Independent and Identically Distributed (non-IID) client data. In the same manner, FedNova averages local updates to eradicate objective inconsistency due to variable local epochs and data scale and fairly participates in clients. They are methods of improving synchronization efficiency and strength in large scale Federated Learning (FL) settings^58,59^.

#### Decision making

**Fig. 4:**
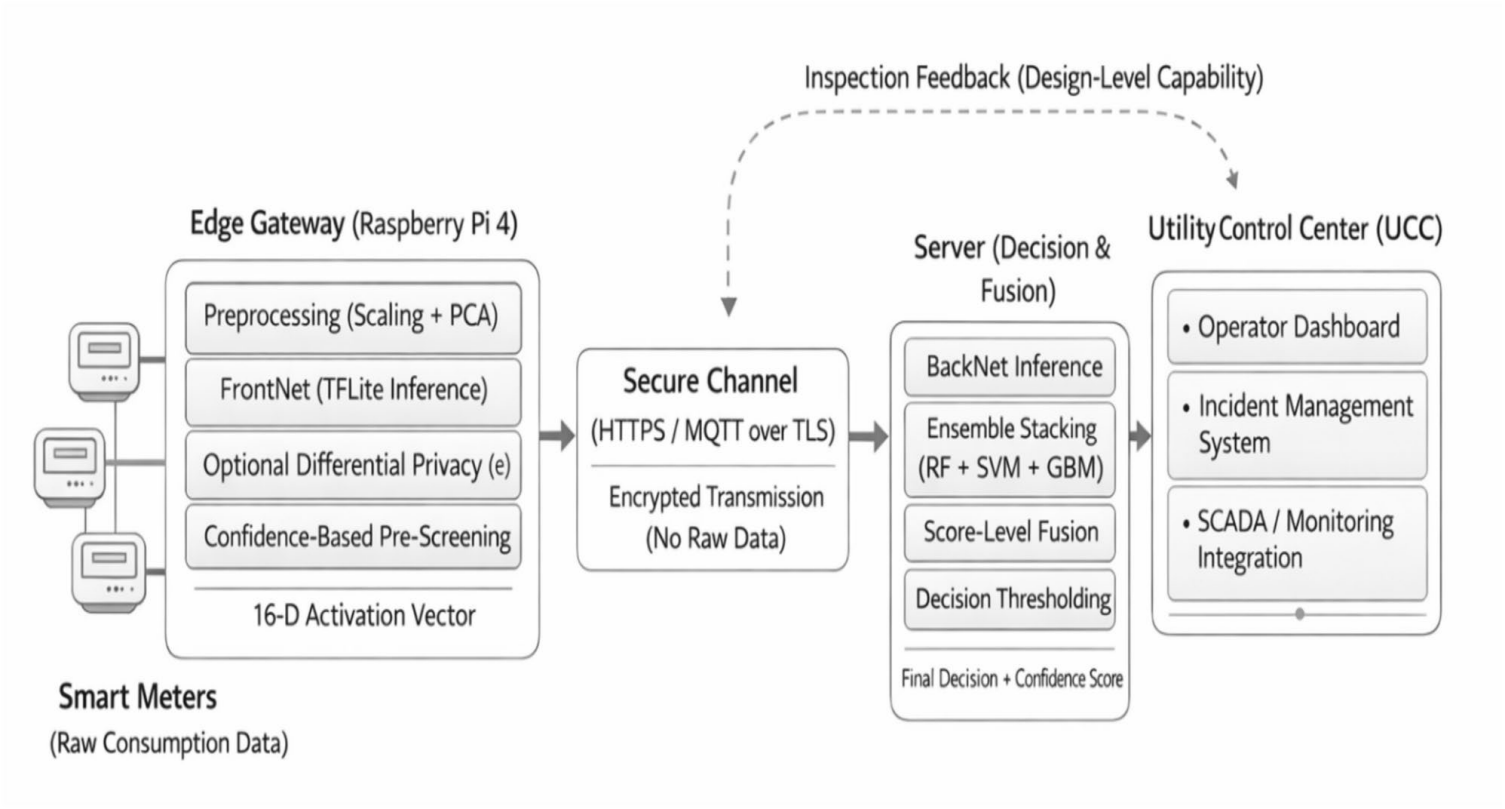
PrivEdge decision-making pipeline across edge and server layers.

After the global model (i.e., the server-side stacking meta-learner) produces its final predictions, each consumer is classified as exhibiting either normal or theft-suspected electricity usage. As illustrated in Fig. 4, PrivEdge adopts a two-tier decision-making pipeline spanning the edge gateway and the server-side inference layers to balance latency, privacy, and decision reliability. Each detected event triggers an alert message that is transmitted to the Utility Control Center (UCC). The alert payload includes the Consumer Identifier (CID), the detection timestamp, and an associated confidence score reflecting the anomaly likelihood. Raw consumption data and high-resolution time-series signals remain stored locally at the edge gateway, while only inference outcomes are communicated.

PrivEdge uses a two-tier decision pipeline to minimize the end-to-end latency, as well as network overhead. At a low-cost stage (first) and threshold-based level pre-screening is performed at the edge gateway (e.g., Raspberry Pi) as a part of the Edge Intelligence (EI) layer. At this step, filtering of obviously normal or highly suspicious patterns is made possible without making any final decisions. Second, confirmation and high-confidence decision making are conducted at the server side, and it involves stacking meta-learner that integrates both temporal and non-temporal signals obtained by BackNet inference and ensemble-based classifiers. This higher-level edge dosage server choice distance minimizes round-trip delay when there are clear anomalies and keeps delicate consumption information home to the meter as is commendable practice in edge-intelligence^60^.

To provide alert delivery and system integration, the architecture depends on recognizing mechanisms based on authenticated and encrypted transport, i.e. Message Queuing Telemetry Transport (MQTT) v5.0 over Transport Layer Security (TLS) or HTTPS/REST with mutual TLS to assure both confidentiality and integrity of the notification channel. Practically generated alerts may be consumed by operational utility systems, such as Supervisory Control and Data Acquisition (SCADA) systems or Incident management structures, to allow them to escalate audits and take steps of follow up by employing Operator responds. Standardized and secure messaging interfaces are supported in interoperability with legacy grid control systems^61^.

The decision-making layer is composed of a number of control mechanisms to attain operational robustness and reduce false positives:

(a) a policy of configurable confidence thresholding, that is, low-confidence warnings are not acted on, but are instead accumulated so that the human operator can look at them. (b) temporal smoothing or non-elasticity of voting in successive inference windows which aim to reduce spikes in the results; and (c) a design with feedback ability where the results of the inspection (true or false positive) can be safely reused in the federated training process to enhance subsequent detection. This kind of hybrid edge-cloud decision strategies are adopted in smart-meter analytics and edge-intelligence systems because of their low-latency and high detection quality when implemented together with grid control infrastructures^10^.

Over time, electricity consumption habits and theft trends can change with seasonal fluctuation, regulatory adjustments or hostile adjustment. PrivEdge uses periodic federated updates to alleviate concept drift and catastrophic forgetting to update its knowledge with a new window of observed data and maintains knowledge learned in the past by performing controlled aggregation and validation-based check pointing. The method allows the model to be trained constantly without the need to gather all the data in a central place or to re-train the model in its entirety.

Lastly, intermittency of client presence and ad hoc network unstable conditions were modeled in training, random subsets of edge nodes were excluded in individual federated rounds, which would represent realistic wide-area conditions of the grid. In these circumstances, the suggested PrivEdge layout had consistent convergence and proscribed inference delay, revealing resilience to only half the number of clients and short-term communication disconnect.

The edge gateway executes lightweight preprocessing and FrontNet inference, producing compact activation vectors and optional confidence-based pre-screening. Privacy-preserving activations are transmitted to the server, where BackNet inference, ensemble stacking, and score-level fusion jointly produce high-confidence decisions. Confirmed alerts are securely forwarded to the Utility Control Center, while optional feedback supports future federated updates.

### Security and privacy protections

**Fig. 5:**
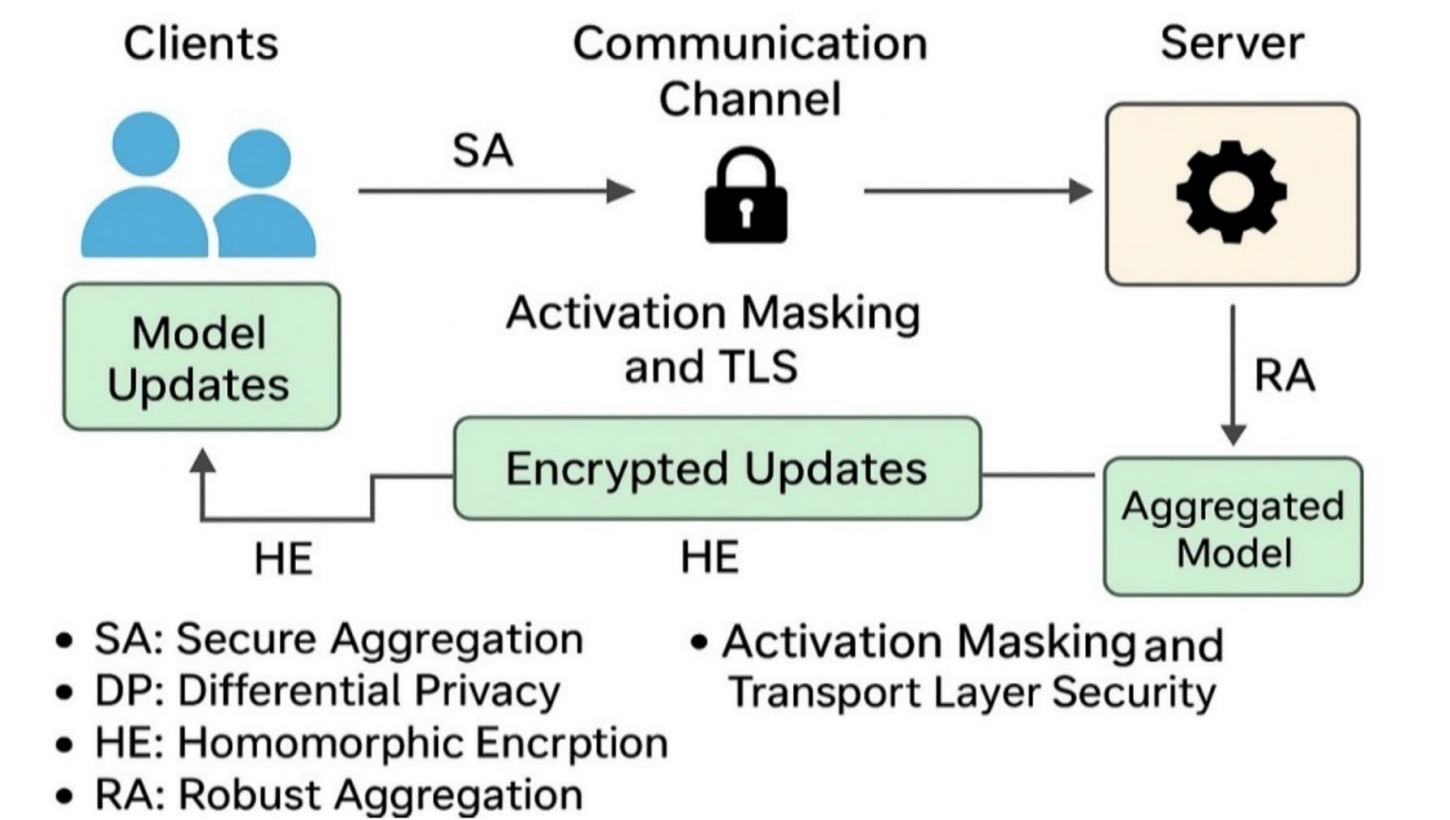
Security and Privacy Protections in the Proposed Framework.

This part is concerned with systems-level security and privacy implications of real-world implementation, as opposed to new cryptographic protocols or adversarial defenses. Some of these mechanisms were discussed briefly above and are listed herein in completeness. In order to guarantee data confidentiality, model integrity, and the ability to combat adversarial behavior, the proposed framework creates several defensive layers as a part of its federated edge cloud architecture. All privacy-saving mechanisms are clearly related to a particular level of the framework either activation transmission, federated aggregation, or secure communication as Fig. 5 demonstrates.

The major protective measures, which are utilized in PrivEdge, can be summed up as follows:

- Secure Aggregation (SA): A cryptography protocol, which allows the server to sum up model updates submitted by involved clients without disclosing the contribution of individual clients, and therefore, maintain local client data privacy in the context of federated learning.
- Differential Privacy (DP): Laplace noise has been introduced on low-dimensional intermediate activations that are conveyed among edge devices and the server. Privacy budget ε is chosen empirically in the domain of 1 ≤ ε ≤ 5. The default operating point is provided as 3 which gives a good trade-off between privacy protection and detection utility with edge constraints in real time unless otherwise. Empirical evidence shows that this setup leads to a small amount of F1-score degradation and the inference-based privacy attack reduction is also high.
- Homomorphic Encryption (HE): HE is considered to be a high-security optional implementation, which enables computation with encrypted data but at extremely high computational and latency. Thus, it is not default enabled during real-time edge deployments and only made to be used in highly sensitive environments that have high regulatory demands. PrivEdge will be constructed in a modular way such that HE can be turned on in case of an increased need of confidentiality guarantees.

There was no experimental analysis of homomorphic encryption carried out in this study, as the reasons are that the computation and latency costs are long known in the literature and intrinsically incompatible with real-time implementation on a Raspberry Pi-class edge device, which is the core of PrivEdge.

- Robust Aggregation (RA): Trimmed Mean and Median aggregation schemes are the Byzantine-resilient aggregation schemes that are used throughout federated BackNet training to reduce the effect of a rogue or malicious client update. These methods help to withhold irregular update values as well as increase defense against model poisoning and backdoor attacks.
- Activation Masking and Transport Layer Security (TLS): Activation masking minimizes the granularity of representations being transmitted to restrict the information leakage and TLS provides encrypted and authenticated communication between edge devices and the server, which cannot be intercepted and modified in transit.

The overheads and computation and communication costs imposed by these security mechanisms are carefully limited. Both secure aggregation and differential privacy have linear time requirements with respect to model size and can be easily handled by Raspberry Pi -like device capabilities. Conversely, homomorphic encryption brings about order-of-magnitude increases in latency and memory and is therefore considered optional. Overall, the combined security stack operates reliably in real time under typical smart-meter sampling rates.

Together, as shown in Figure 5 and summarized in Table 3, these complementary mechanisms establish a privacy-preserving and attack-resilient learning environment, making the proposed framework well suited for real-world smart-grid and IoT-based electricity theft detection deployments^62^.

**Table 3:** Comparison of privacy-preserving mechanisms in PrivEdge.

Mechanism	Privacy level	Computational overhead	Communication overhead	Real-time suitability
Secure aggregation	Medium–High	Low	Low	✔
Differential privacy	High (ε-bounded)	Low–Medium	Low	✔
Homomorphic encryption	Very High	High	High	✖ (Optional)

These complementary mechanisms, as shown in Fig. 5 and summarized in Table 3, offer in combination a privacy preserving and attack resistant learning environment. In contrast to previous work which considers privacy mechanisms as independent entities, PrivEdge integrates security and privacy safeguards into the hybrid SL–FL workflow, through which implicit end to end protection is achieved during real-time edge execution. Notably, the privacy leakage issue is not just a conceptual discourse, yet an empirical quantification of the issue is done using empirical membership inference and model inversion attacks, under realistic black-box adversarial conditions, as described in Section "Empirical evaluation of privacy attacks".

### Edge implementation on Raspberry Pi 4 model B

The proposed system deploys are optimized for resource-constrained edge devices, the Raspberry Pi 4 Model B in particular, to which there is a good balance between computational performance and energy efficiency and manageable cost. A number of optimization strategies were applied in order to facilitate the on-device intelligent capability.

To start with, trained deep learning models are then transformed into either TensorFlow Lite (TFLite) or ONNX format which are popular in lightweight inference on embedded and edge platforms because they can be executed with lower computational overhead and with portability^37,63^. Then, we implement quantization and pruning algorithms to reduce the size of the neural network and its memory footprint as well as inference latency by almost half, yet maintain the accuracy^64,65^. Furthermore, hardware acceleration is made possible by devices like the Google Coral TPU or Intel Neural Compute Stick 2 (NCS2) using optional modules that deliver increased processing speed and decreased power usage for real-time processing^65,66^. Lastly, multithreading based parallel processing architecture is embraced to process preprocessing, model inference and communication functions at the same time, to offer real-time responsiveness and high throughput through the entire edge pipeline^37,63^.

It should be pointed out that the suggested PrivEdge architecture is devised as a scalable and composite framework. Although every component (PCA, SMOTE, Split Learning, Federated Learning, and ensemble stacking) is activated in the whole system, all the modules can be activated/deactivated despite deployment constraints, privacy needs, and the availability of edge resources. This modularity eases the cost of operation and gives it flexibility in adaptations to heterogeneous smart grid environments.

Although extreme multi-day stress scenarios and forced crash recovery were not explicitly emulated, the observed absence of memory drift, thermal throttling, or runtime instability over 24 h provides strong evidence of operational robustness for practical deployments.

## Results

### Dataset description

The experiments conducted in this research were done using the data set of the State grid corporation of China (SGCC)^8^ which is a broad data on electricity consumption among a vast variety of residential, commercial and industrial clients. All of these data are in the form of normal use and errors of non-technical nature (electricity theft), which is why this data is applicable in creating and testing models of electricity theft detection. The major attributes of the SGCC data are:

- Scale: Tens of thousands of customers have hourly electricity readings over the course of several months.
- Features: Contains raw data of consumption as well as computed features like time of use patterns, variability of loads, and aggregate consumption statistics.
- Labeling: The consumption records are labeled as either normal or fraudulent, which allows supervised learning strategies.
- Challenges: The data set is very lopsided, as there are significantly fewer cases of theft than the cases of normal use, thus necessitating the use of class balancing methods like SMOTE.

The dataset gives a realistic situation to test advanced machine learning models, such as Edge AI, federated learning (FL), and split learning (SL), and it is possible to assess both the predictive accuracy and the effectiveness of communication in edge deployment situations.

As usual in previous research in the electricity theft detection hybrid, we utilize the public dataset of SGCC with preprocessing and labeling configuration as in the prior research^8^, in order to provide fair and reproducible comparison between the centralized and distributed learning paradigms.

To reduce data leakage as well as idealistic performance estimates in all experiments, the dataset was divided at the customer level so that the consumption records of the same customer cannot be found in both training and testing sets.

Even though experimental validation is based on the use of the SGCC dataset, the dataset is commonly used as a benchmark across electricity theft detection and captures long-term effects of consumption, class imbalance, and realistic noise effects. The shallower model of PrivEdge framework is data-source clean with respect to data sources and can be easily modified to other regional data in smart grids by changing window sizes, sampling frequencies, and preprocessing options. It will be extended to validation of across-dataset with other public and utility-provided datasets in the future to further determine the generalizability of the results with other grid structures and theft patterns.

### Experimental setup

The suggested framework was tested on the platform of Raspberry Pi 4 model B, which is a common resource-limited edge device. The offline training and ablation experiments were performed in a controlled setting whereas on-device experiments were made to concentrate on real-time inference, communication efficiency, and edge-side resource consumption to be in line with realistic deployment settings.

In order to deploy on the edge, the trained FrontNet model was converted to TensorFlow Lite (TFLite) and ran on the Raspberry Pi 4 at low latency split inference. The feature normalization and dimensionality reduction were used in all the experiments and no raw consumption data were transferred further than the edge device.

During the federated training stage, a fixed strategy of client participation was used with all available clients participating in every training round except when mentioned otherwise. Every client conducted one local training round with the same optimizer and learning rate in Table 4. Such an arrangement was chosen as it is a realistic environment of smart-grid deployment, in numerous ways where periodic synchronization, and little local computation is favored over local long-term training.

**Table 4:** Training and deployment configuration for Exp-3.

Component	Configuration
Dataset	SGCC smart meter dataset
Original feature dimension	1034
Feature normalization	StandardScaler
Dimensionality reduction	PCA (200 components)
Class balancing	SMOTE (k = 1)
Random seed	42
FrontNet architecture	Dense layers [256, 128] → 16-D output
BackNet architecture	Dense layers [128, 64] → sigmoid
Optimizer	Adam
Learning rate	1 × 10⁻^3^
Max training epochs	80
Early stopping	Patience = 10 (PR-AUC)
Training batch size	512
Client participation per FL round	100% (all available clients)
Local epochs per client	1
Split point	After FrontNet (16-D activations)
Ensemble models	RF (300 trees), SVM, GBM
Meta-learner	Logistic Regression
Fusion strategy	Linear score fusion (α = 0.5)
Edge inference	TFLite FrontNet on Raspberry Pi 4
Edge batch size	64
Differential privacy	Laplace DP on activations (ε = 1,3,5)
Secure aggregation	Server-side aggregation
Homomorphic encryption	Not enabled in Exp-3
Decision thresholds	Selected by best F1 on validation set

The experimental comparison will also involve centralized deep learning (DL), federated learning (FL), split learning (SL), and the hybrid SL–FL construction suggested in this study. Each of the models was tested by the standard classification measures such as Accuracy, Precision, Recall, and F1-score, as well as communication-related measures that indicate the overhead of the collaborative learning.

All the baseline models were compared on the same preprocessing, data partitioning, and hardware settings to achieve consistent comparison. In case it was feasible, baseline implementation was redone in the same experimental setup. In the case of hybrid frameworks, whose full implementations were not available, results reported in the original studies were mentioned explicitly and differentiated clearly between reproduced and original.

Experiments with deployment were continuously executed on the edge and server components and all the reported performance, latency and resource metrics were based on recorded execution logs and not simulations or estimates.

Although the experiment analysis is performed on the SGCC dataset which is extensively used. The proposed framework is not data-source dependent in electricity theft detection. Cross-dataset generalization is therefore left as future work, as explicitly acknowledged in Section "Conclusion".

#### Experimental configuration and reproducibility

To achieve complete experimental transparency and reproducibility, all training, deployment, and inference parameters of the proposed PrivEdge framework were set in fixed and consistent values and used throughout the experimental pipeline. This consists of data preprocessing, model architecture, model optimization parameters, privacy enablers, and decision threshold. Table 4 highlights the entire setup of the full end-to-end deployment scenario (Exp-3) which is the most realistic and all-inclusive evaluation environment. Any parameter that is listed in the table is an actual implemented parameter utilized in both training and hardware-in-the-loop deployment to Raspberry Pi 4 and the server, without any reference to estimation, simulation, or post hoc tuning.

All federated round involves complete client participation of one local epoch per client to guarantee the case of stable convergence and reproducibility of one of these closely regulated through deployment constraints.

The training, edge deployment, and server-side inference code are all implemented and match all configurations in Table 4. There are no estimated or simulated parameters, and all values are obtained by performed experiments.

### Performance metrics

The following metrics were used to evaluate the proposed model and comparative approaches:

- **Accuracy:** The percentage of the properly classified instances among all instances.
- **Precision:** The percentage of successful predictions of thefts in all predicted thefts, the measure of the reliability of the model to predict theft.
- **Recall (Sensitivity):** The percentage of real thefts that were correctly identified and this is the measure of the ability of the model to detect a theft without false alarms.
- **F1-Score:** The harmonic average of precision and recall, which is a balanced measure with specific importance in application using imbalanced data.
- **Cost of Communication:** The amount of data transferred between edge devices and central servers, which is essential in the EDGE computing case where there is a constraint on bandwidth and latency.

All of these metrics make an ultimate analysis of detection performance and operational effectiveness in edge environments^67^. Along with accuracy, precision and recall as well as F1-score, the suggested framework is also assessed with respect to Area Under the Receiver Operating Characteristic Curve (AUC-ROC) and Precision-Recall (PR) curves. These measures are especially appropriate in the case of electricity theft, where the imbalance of classes is natural, and false negatives may be extremely costly to operate. The findings suggest that PrivEdge has a better AUC-ROC and PR performance than centralized, FL-only, and SL-only baselines in all studied scenarios and has a greater ability to distinguish in the presence of class imbalance.

### Experimental results

#### Detection performance results

The results of detection performance of Experiment 3 representing the complete PrivEdge deployment scenario have been reported in this subsection. This experiment uses the full pipeline of the edge-to-server inference system, on-device preprocessing, split (Raspberry Pi) inference, and BackNet inference server-side, ensemble stacking, and score-level fusing. Any reported measures are obtained from the experimented metrics and inferred log of experiments, but not offline simulation.

The proposed model was tested with respect to various state-of-the-arts models, such as centralized deep learning (DL), federated learning (FL), split learning (SL), and hybrid FL-SL models. These evaluation metrics are Accuracy, Precision, Recall, F1-Score and Communication Cost that indicate not only performance in predictions but also the cost of predicting edge deploys ability. In Table 5, the results are summarized.

**Table 5:** Detection performance comparison under Experiment 3 (full PrivEdge deployment).

Approach	Model	Accuracy (%)	Precision (%)	Recall (%)	F1-Score (%)	Communication Cost
Centralized DL	CNN^9^)	96.0	95.7	96.3	96.0	High
FL	Hybrid DL—FL^5^)	95.8	95.4	95.9	95.6	Medium
FL	Deep Anomaly Detection—FL^10^)	96.5	96.0	96.8	96.4	Medium
FL	HeteroFL (CNN-LSTM—CKKS)^11^	97.0	96.6	97.3	96.9	Medium
SL	SL + Demand-Response^12^	95.0	94.5	95.2	94.8	Low–Medium
SL	Transformer-SL—GAN—ECDH^13^	96.2	95.8	96.4	96.1	Low–Medium
Hybrid FL -SL	Edge-assisted U-Shaped FL—SL^7^	97.3	97.0	97.5	97.2	Low
Hybrid FL -SL	FUSE Hybrid FL—SL^14^	97.8	97.5	98.0	97.7	Low
Hybrid FL -SL	Proposed Model (PrivEdge – PCA + SMOTE + Stacking + Fusion)	98.06	97.82	98.31	98.06	Low

Reported results for the proposed model correspond to the No-DP reference configuration (upper-bound performance), while privacy-aware variants are analyzed separately in Section "Privacy–utility–latency trade-off".

Otherwise, the baseline models were compared under a similar preprocessing pipeline, data division scheme, and hardware requirements with the aim of making a fair comparison. Models whose complete implementations could be obtained were replicated in the same experimental set up. In hybrid frameworks, wherein it was not possible to have full reproduction, the reported results of the original studies were used directly and compared using similar evaluation measures.

The proposed PrivEdge framework, as demonstrated by Table 5, has the best overall performance in terms of detection performance and a low communication overhead, which means it can easily be deployed to resource-constrained edge devices like the Raspberry Pi 4. These positive performance changes are explained by the synergistic effect of localized preprocessing, hybrid split -federated inference and decision fusion through ensembles instead of the use of a single modeling component. This could be also observed on the basis of ablation analysis in Section "Ablation study".

In order to determine the reproducibility of the noticed improvements in performance, statistical significance was determined by evaluating PrivEdge in comparison with the best baseline models based on repeated inference runs with constant decision thresholds. To ensure robustness, we repeated inference evaluation using fixed decision thresholds and observed consistent performance trends across runs.

*Sensitivity to different electricity theft patterns*: The heterogeneous behavior patterns of electricity theft include sustained low amplitude theft (e.g. a sustained pattern of under-reporting consumption) and intermittent high amplitude theft (e.g. intermittent large consumption manipulation). Even though the SGCC dataset does not directly indicate the theft occurrences in terms of behavioral subtype, previous research has determined these patterns are implicitly represented in the temporal consumption dynamics and variability measures.

PrivEdge has been architecturally and model-designed to elicit this range of heterogeneous theft behaviors. The FrontNet component trains compact temporal structures that focus on the regularity, volatility and long-term deviation of consumption, and thus it is susceptible to small anomalies that prevail over a longer period and could be missed by threshold-based detectors. In the meantime, ensemble stacking and BackNet inference are more sensitive to intermittent large-scale theft (when sudden deviations are prevalent in the short run).

This can be empirically observed by the high Recall (98.31%) and ROC-AUC (≈0.99) obtained in Table 5, which shows that both subtle and prominent cases of thefts are localized with high levels of confidence. Remarkably, the high recall would imply that the framework hardly overlooks theft events, and this is especially important in low-amplitude persistent cases of theft that incur huge losses with time.

Moreover, its stability over a wide range of behavioral regimes is indirectly confirmed by the strength of PrivEdge against poisoning attacks (Section "Security discussion: robustness against poisoning attacks"). The detection performance does not significantly decrease when the labelling or feature scale is manipulated by the malicious clients, and it is likely that the learned representations are not highly specialized to a single theft motif but instead that they generalize across several structures of anomalies.

In general, even though the explicit subtype labelling falls outside of the framework provided by the presented dataset, the overall architectural design and the empirical performance measure prove that PrivEdge is capable of ensuring a high detection efficacy over a broad range of realistic electricity theft behaviors.

#### System overhead, privacy trade-off, scalability, and practical feasibility analysis

While the preceding sections demonstrated the strong detection capability of the proposed PrivEdge framework, real-world deployment in smart-grid environments necessitates a system-level evaluation that extends beyond predictive accuracy alone. In practice, edge-based electricity theft detection systems must satisfy strict constraints on computational load, memory usage, communication overhead, inference latency, energy consumption, scalability, and long-term operational stability—particularly when privacy-preserving mechanisms are integrated.

This section presents a comprehensive system-level assessment of PrivEdge based on continuous 24-h executions on real hardware. A Raspberry Pi 4 Model B was employed as a representative edge gateway, while an Arduino-based metering layer was used to emulate field-level smart meters. Three experimental configurations were designed and evaluated to progressively assess architectural complexity, privacy integration, and deployment realism.

##### Experimental configurations and deployment scenarios

To enable a transparent and structured system-level evaluation, three experimental configurations were designed to incrementally increase system complexity and deployment realism. Each experiment isolates a specific architectural aspect of PrivEdge, allowing the impact of (i) edge-only inference, (ii) privacy-aware split inference, and (iii) full hardware-in-the-loop deployment to be assessed independently and comparatively.

**Fig. 6:**
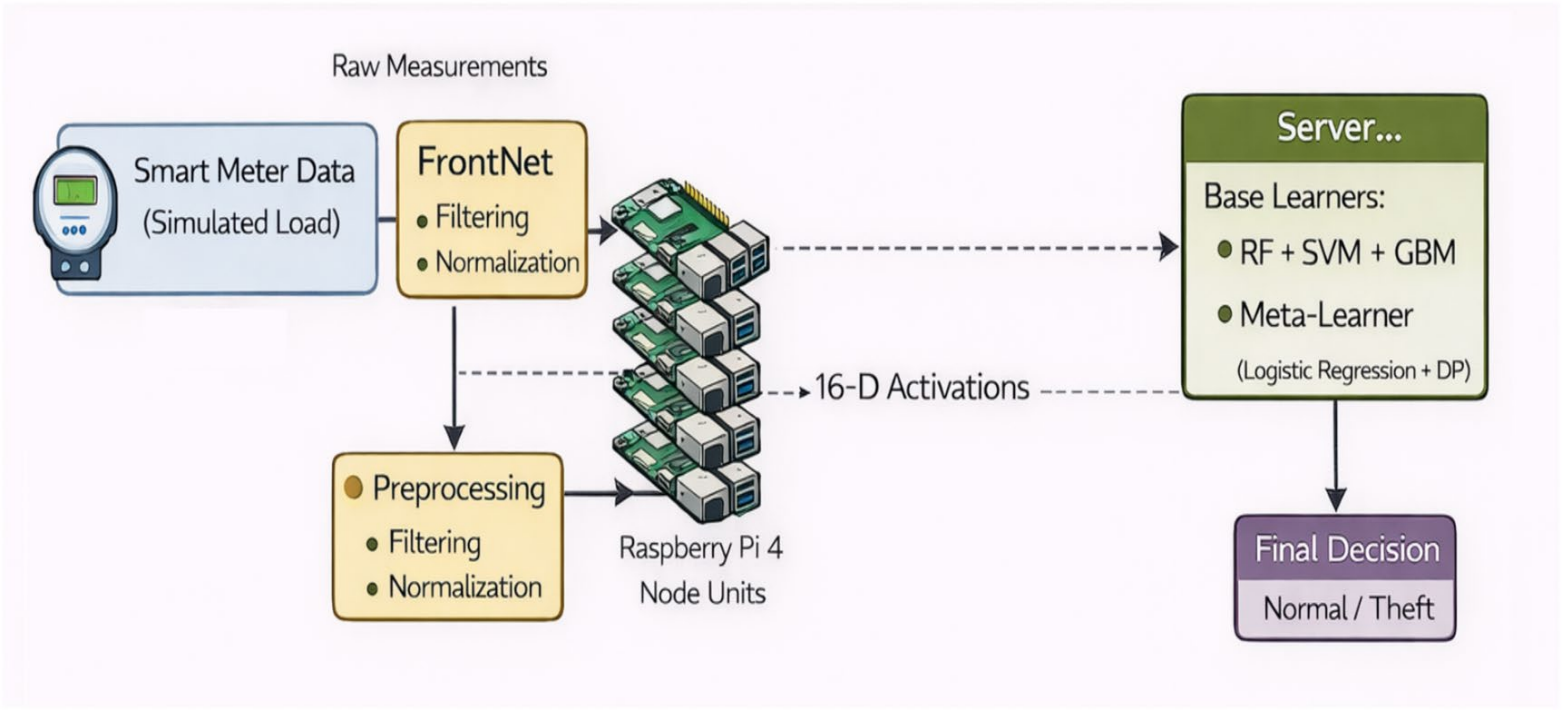
Experiment 1 Edge only Inference with ServerSide Stacking (Baseline).

*Experiment 1 (Exp-1): Edge-only inference with server-side stacking (baseline)*. Exp-1 establishes a lightweight baseline by running only the FrontNet inference on the Raspberry Pi (TFLite), then transmitting the resulting compact representations to the server where a classical stacking ensemble performs classification. This experiment intentionally excludes BackNet and fusion, as well as the Arduino metering layer, to isolate the intrinsic overhead of the edge inference pipeline and server-side stacking. The overall architecture of this baseline configuration is illustrated in Fig. 6.

**Fig. 7:**
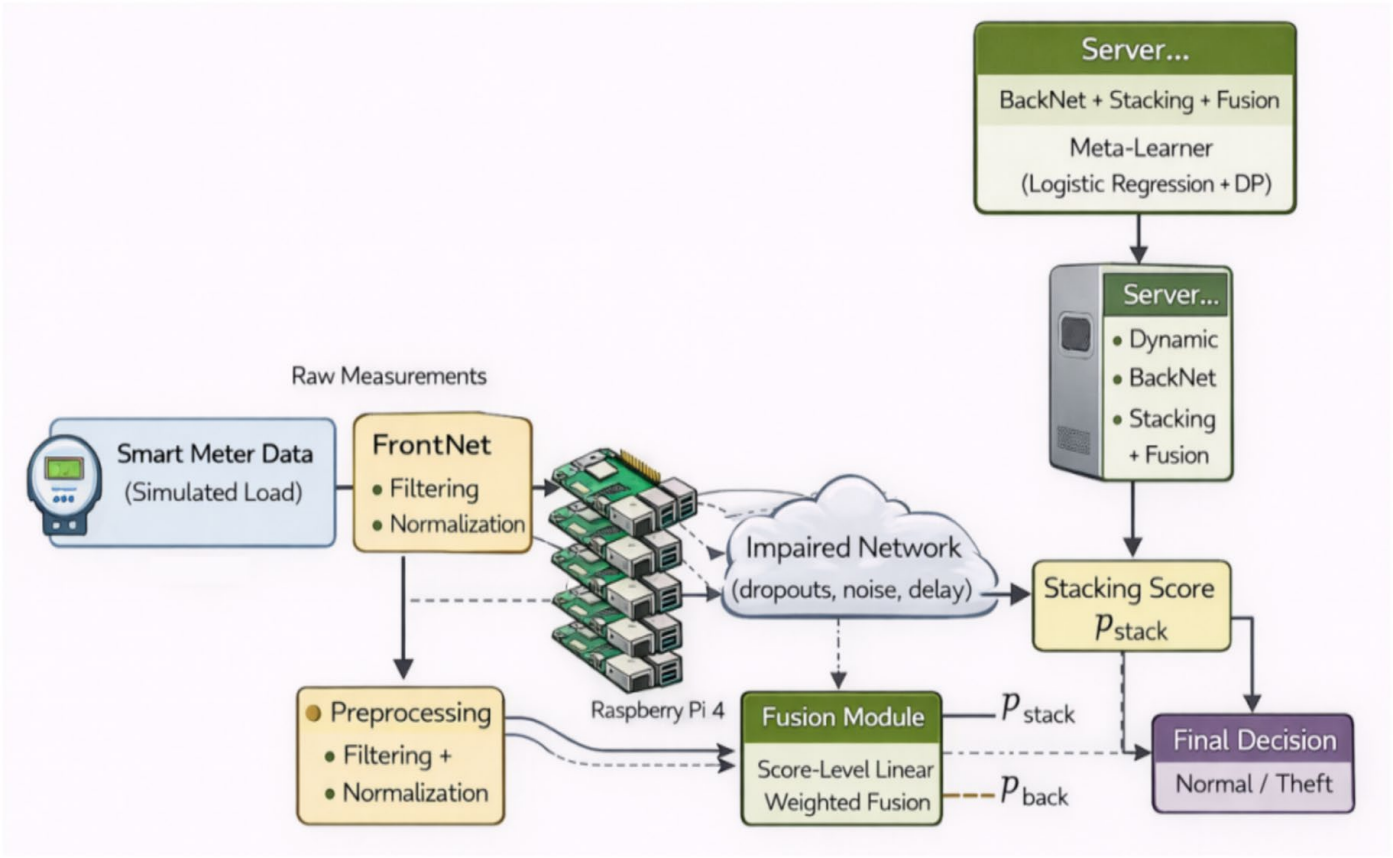
Experiment 2 (Exp-2): Privacy-aware split inference with BackNet, stacking, score-level fusion, and differential privacy under controlled network conditions.

*Experiment 2 (Exp-2): Privacy-aware split inference with BackNet and stacking*: Exp-2 activates the full split inference workflow: the Raspberry Pi executes FrontNet and applies Laplace Differential Privacy (DP) on the intermediate activations. The server performs BackNet inference, stacking, and score-level fusion. This configuration allows controlled evaluation of the privacy–utility trade-off under different privacy budgets (ε) while maintaining a deployment pipeline consistent with PrivEdge operation. Figure 7 illustrates the privacy-aware split inference workflow adopted in Exp-2.

**Fig. 8:**
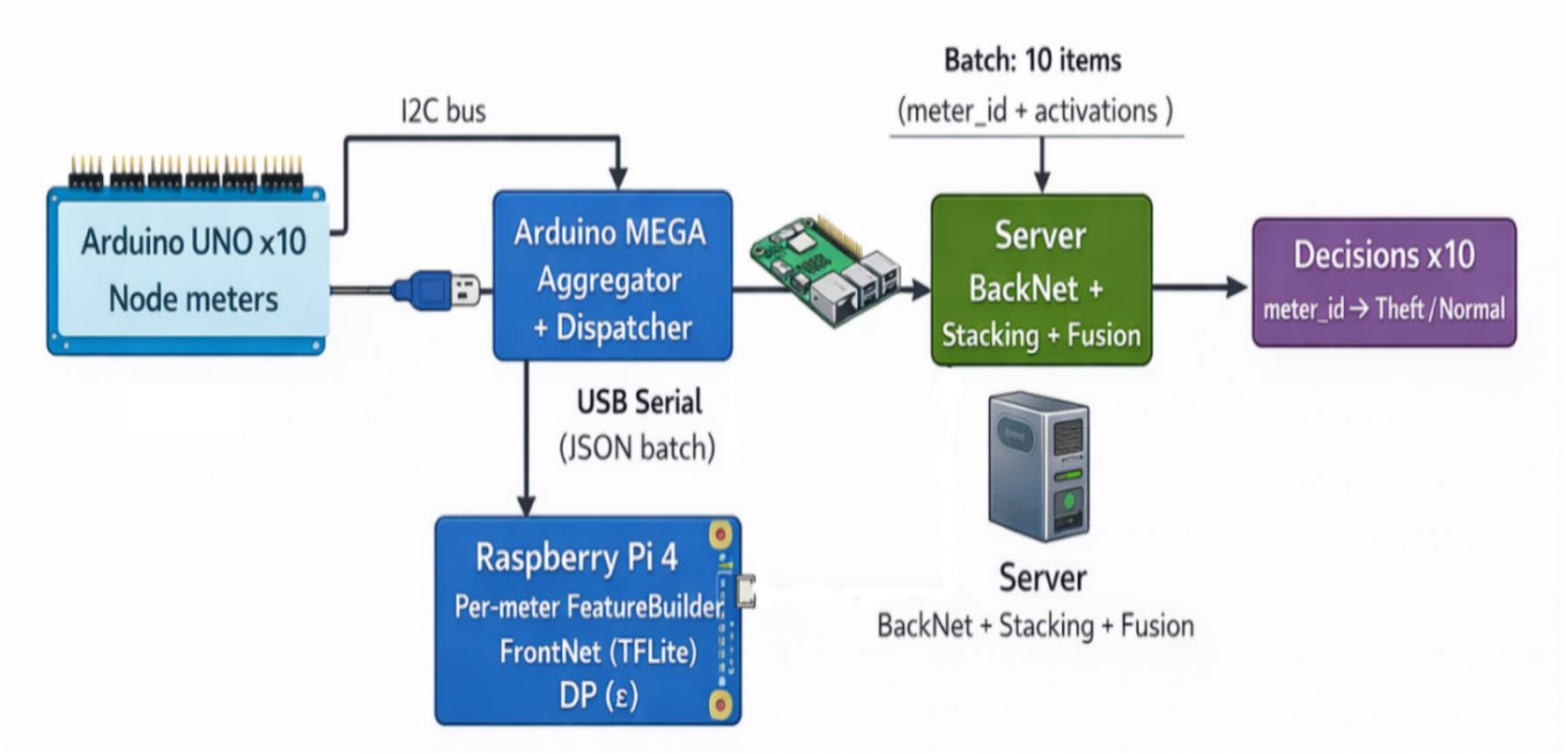
Experiment 3 Full end-to-end deployment with Arduino integration and fusion.

**Fig. 9:**
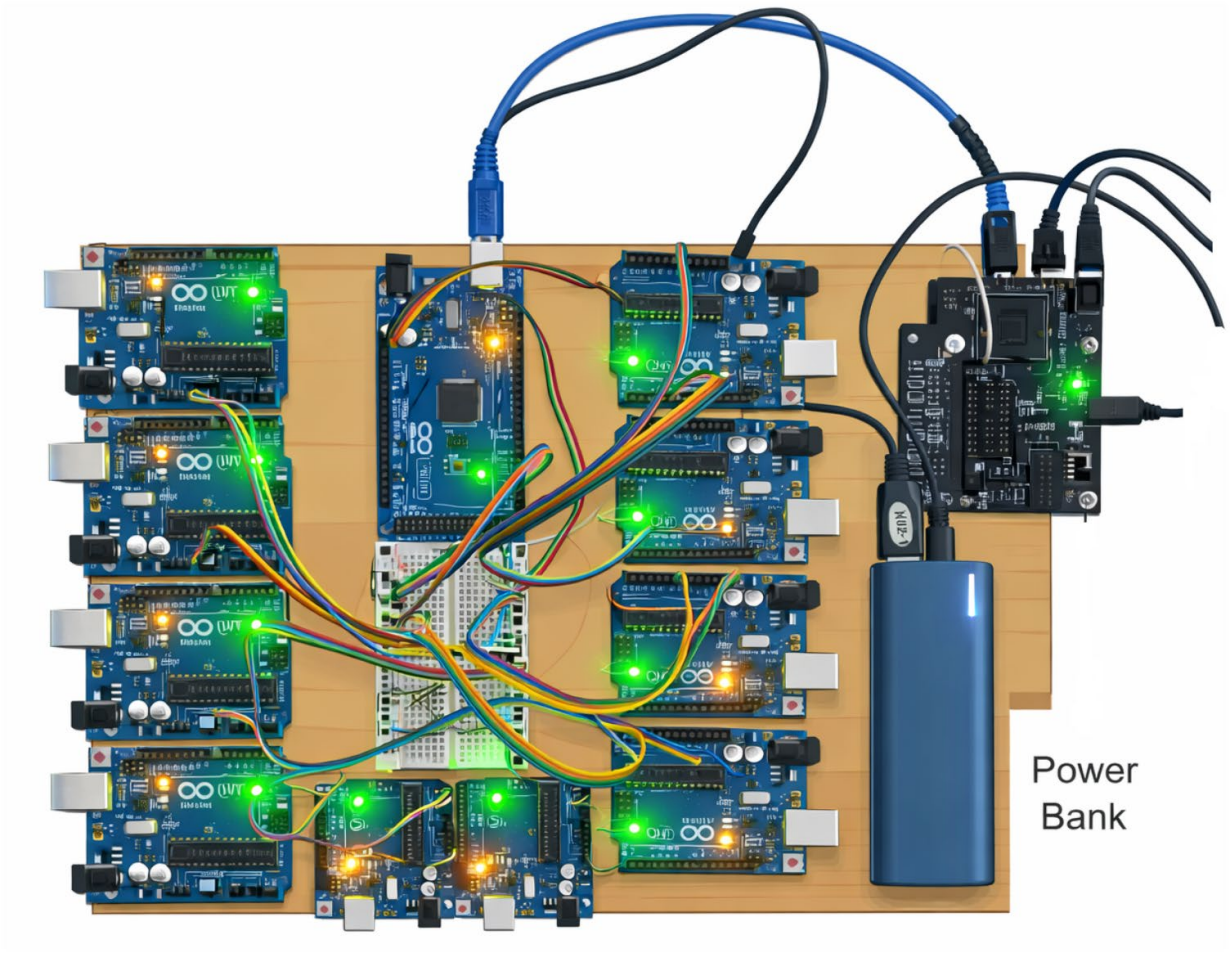
Experimental hardware setup of Experiment 3 (distributed multi-node edge sensing architecture).

*Experiment 3 (Exp-3): Full end-to-end deployment with Arduino integration and fusion*: Exp-3 represents a realistic multi-node IoT scenario. Ten Arduino Uno boards emulate independent smart meters, aggregated via Arduino Mega (I^2^C), with serial ingestion into the Raspberry Pi (USB). The Raspberry Pi performs feature building and FrontNet inference, optionally applies DP, and transmits activations in batch (size = 10) to the server. The server performs BackNet inference, stacking, and score-level fusion to produce per-meter decisions. This experiment is the closest to field deployment conditions and is used to assess end-to-end latency, throughput behavior, and long-term stability under sustained workload. The full end-to-end deployment scenario of Exp-3 is depicted in Fig. 8. The corresponding hardware setup for Exp-3 is shown in Fig. 9.

Table 6 summarizes the key characteristics of the three experimental configurations.

**Table 6:** Summary of experimental configurations.

Experiment	Edge configuration	Server components	Privacy mechanism	Hardware setup	Duration
Exp-1	FrontNet (TFLite)	Stacking only	None	Raspberry Pi 4 + Server	24 h
Exp-2	FrontNet + Differential Privacy	BackNet + Stacking + Fusion	Laplace DP (ε-controlled)	Raspberry Pi 4 + Server	24 h
Exp-3	FrontNet + Differential Privacy + Serial Ingestion	BackNet + Stacking + Fusion	Laplace DP (ε-controlled)	Arduino UNO × 10 + MEGA + Raspberry Pi 4 + Server	24 h

All experiments were executed continuously for 24 h to capture sustained workload behavior, transient resource utilization peaks, and long-term operational stability.

##### Contribution of BackNet and Fusion components (Incremental analysis)

Relevant contrib. of individual architectural parts revealt individual in the PrivEdge framework is made by incremental comparing the three inference configurations that represent the different experimental configurations introduced in Section "Experimental configurations and deployment scenarios". The goal of this analysis is to isolate the influence of BackNet-enabled split inference and score-level fusion and to ensure that the gains have been attributed to, and are not related to results from, unnecessary complexity of the system.

In particular, the following inference paths are subject to the same preprocessing process, data partitions and decision threshold:

1. Stacking only inference (Exp-1): where compact activate vectors created by the edge-side of FrontNet is classified with the ensemble stacking model on server.
2. Stacking + BackNet inference (Exp-2) On top of split inference in the split activation, we introduce server-side BackNet without removing the ensemble of stacking.
3. Stacking + BackNet + Fusion (Exp-3) which is full PrivEdge deployment, where the BackNet outputs and the stacking ones are both used through score level fusion.

Table 7 summarizes the detection performance across the three incremental PrivEdge inference configurations.

**Table 7:** Detection performance across incremental PrivEdge inference configurations.

Inference Configuration	Accuracy (%)	Precision (%)	Recall (%)	F1-score (%)
Stacking only (Exp-1)	98.10	98.04	98.18	98.11
Stacking + BackNet (Exp-2)	97.92	97.88	97.98	97.93
Stacking + BackNet + Fusion (Exp-3)	98.06	97.82	98.31	98.06

*All results are obtained under the same preprocessing pipeline and evaluation protocol. Reported values correspond to the no-DP reference configuration to isolate architectural effects.

Table 7 shows that stacking-only inference offers a high-quality baseline and with a low degree of architectural complexity. By adding BackNet in Exp-2, we can learn more about more time-related information by split inference, which leads to competitive detection results with keeping privacy-oriented system characteristics. The entire PrivEdge set up in Exp-3 has the greatest overall robustness through the use of complementary decision signal of BackNet and ensemble stacking via score-level fusion.

Such results affirm that the enhancement in performance that PrivEdge shows is due to the synergistic action between split learning, ensemble stacking and fusion and not due to any individual element alone. Notably, the incremental gains are realized without the need to add restrictive system overhead, which justifies the design decisions in the architectural design chosen in the entire PrivEdge deployment.

##### Edge computation, memory, and energy overhead

**Fig. 10:**
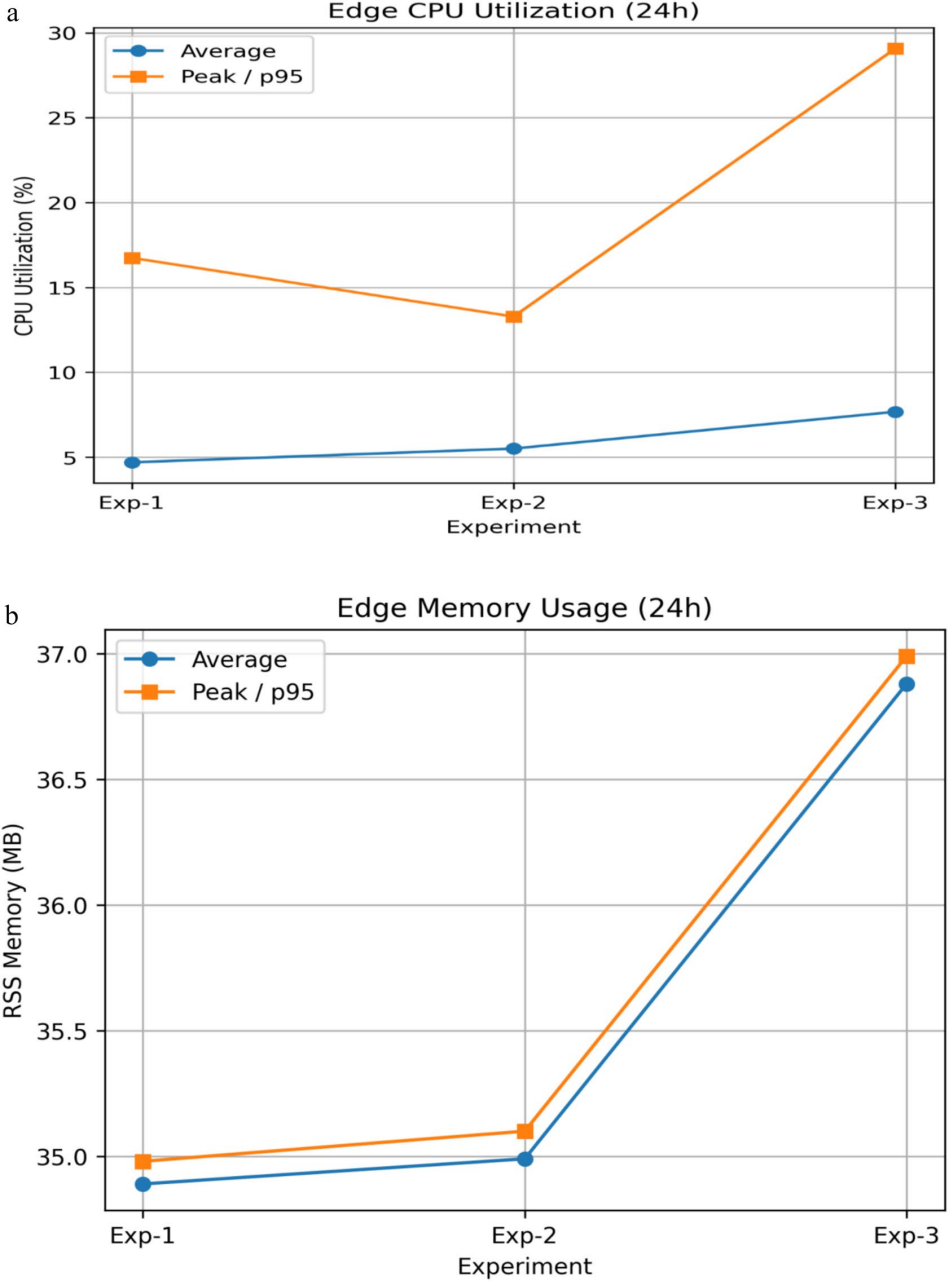

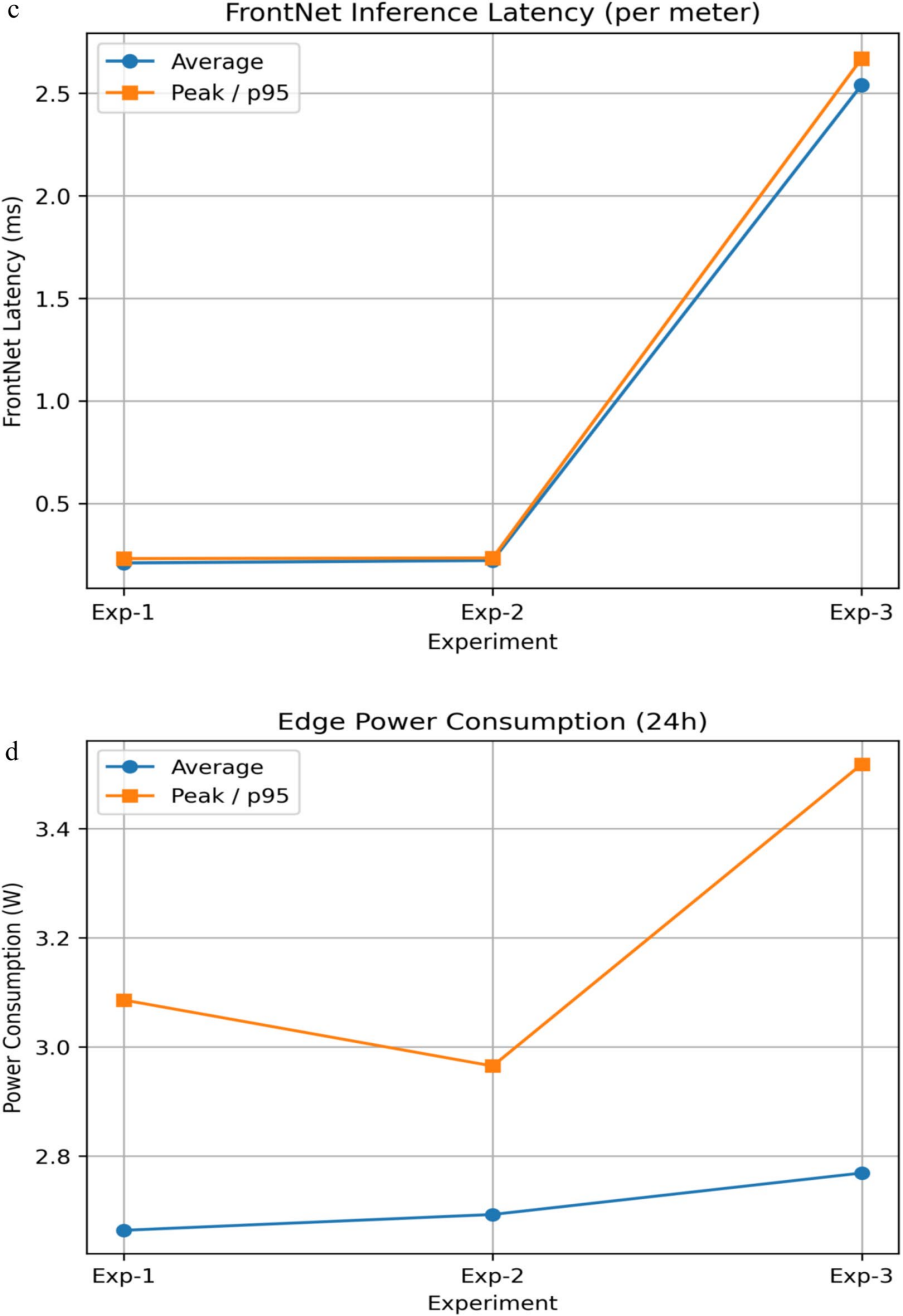

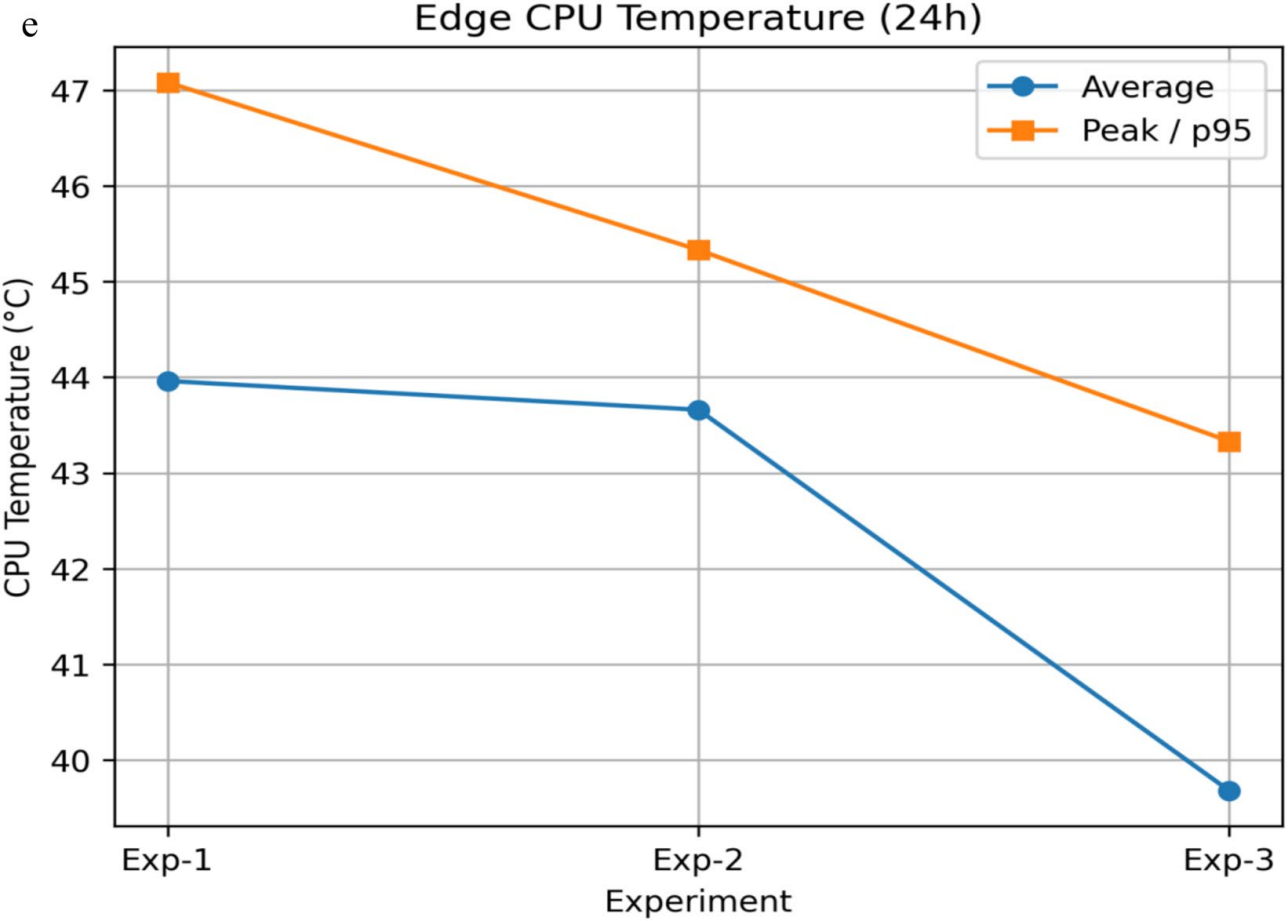
Edge-side resource behavior over 24 h across Exp-1, Exp-2, and Exp-3. (**a**) Edge CPU Utilization Across Experiments. (**b**) Edge memory usage (RSS) across experiments. (**c**) FrontNet Inference Latency (per meter). (**d**) Edge Power Consumption Across Experiments. (**e**) CPU Temperature Across Experiments.

Figure 10 illustrates the edge-side resource behavior of the Raspberry Pi 4 over 24 h across Exp-1, Exp-2, and Exp-3, including (a) CPU utilization, (b) RSS memory usage, (c) FrontNet inference latency per meter, (d) power consumption, and (e) CPU temperature. The results demonstrate stable long-term execution with limited overhead despite increased system complexity. In this subsection, Table 8 reports sample-level instantaneous measurements (collected periodically during execution) to capture transient peaks and variability. These statistics reflect the distribution of sampled points over 24 h rather than window-aggregated stability metrics.

**Table 8:** Instantaneous edge-side resource utilization and peak load characteristics (24-h execution on Raspberry Pi 4).

Metric		Exp-1	Exp-2	Exp-3
Avg CPU (%)		4.69	5.50	7.67
Peak CPU (%)		16.74	13.28	29.08
CPU std (%)		1.51	1.39	2.85
Avg RSS (MB)		34.89	34.99	36.88
Peak RSS (MB)		34.98	35.10	36.99
RSS drift (MB)		0.35	0.38	0.65
Avg FrontNet latency (ms)		0.209	0.221	2.540 *(per meter)*
p95 FrontNet latency (ms)		0.230	0.233	2.669 *(per meter)*
Avg power (W)		2.664	2.693	2.769
Peak power (W)		3.086	2.965	3.518
Avg CPU temp (°C)		43.96	43.66	39.68
Peak CPU temp (°C)		47.08	45.33	43.33

The baseline configuration (Exp-1) shows very low average CPU utilization and stable RAM usage, reflecting the lightweight nature of FrontNet execution on Raspberry Pi.

In Exp-2, adding DP does not materially increase average resource consumption, confirming that privacy injection on intermediate representations is computationally feasible on resource-constrained gateways.

In Exp-3, the increase in memory footprint is expected due to serial ingestion, per-meter feature construction, and batch handling of 10 m. Despite this, the edge power consumption remains stable, and thermal measurements stay within safe limits. These results collectively confirm that PrivEdge can operate continuously on Raspberry Pi hardware without resource exhaustion.

It should be noted that these metrics represent instantaneous and peak behavior, and therefore may differ from long-term aggregated statistics reported in Section "Long-term operational stability", which focus on operational stability over extended execution periods.

In this paper, battery life was not specifically tested because Raspberry Pi-based edge computers in smart-grids are usually not battery-powered but mains-powered. As a result, average and peak power consumption measures are deemed apt measures of energy efficiency in operations within the target deployment environment.

*Implications of battery-powered deployment*: Even though the present experimental platform is fed through a fixed external source, the given power measurements contain valuable information on the possibility of deploying it using the battery. The reported mean power consumption of the Raspberry Pi 4 when sustained and running in Exp-3 is 2.66 to 2.77 W, with peak values remaining below 3.6 W.

In standard battery-powered IoT implementations (i.e. a 10,000 mAh, 5 V, battery pack, or which is equivalent to approximately 50 Wh) the power profile would be observed to translate to a continuous operation time of at least 14–18 h, based on workload intensity and communication frequency. Notably, PrivEdge is event-driven and batch based, as opposed to continuous pervasive sensing that operates at high-frequency, and this further increases useful battery life in operational systems.

In addition, the lack of thermal throttling, constant CPU usage, and limited power variation recorded in the 24-h test execution of PrivEdge suggests that the device is not triggering power spikes that can speed up battery decay or negate the stability of the device. These features are especially applicable in case of gateway-class edge nodes that are used in remote or infrastructure constrained smart-grid environments.

Although battery discharge profiling cannot be accurately performed in this work, it has been established that PrivEdge can still be deployed with battery-assisted/hybrid-powered edges and is consistent with the operational limits of smart-grids in reality.

##### Server-side computation overhead

Server-side behavior was evaluated under continuous execution, focusing on (i) inference pipeline complexity, (ii) latency contribution of BackNet/stacking/fusion, and (iii) stability under sustained load.

Table 9 summarizes the server-side computation characteristics observed during 24-h continuous execution across the three experimental configurations.

**Table 9:** Server-side computation characteristics (24-h execution).

Metric	Exp-1	Exp-2	Exp-3
BackNet enabled	No	Yes	Yes
Stacking enabled	Yes	Yes	Yes
Fusion enabled	No	Yes	Yes
Batch size	1	1	10
Performance degradation over time	No	No	No

In the baseline configuration (Exp-1), server-side computation is limited to stacking-based inference on compact activation vectors, resulting in minimal computational overhead. The introduction of BackNet in Exp-2 increases inference complexity as expected; however, continuous monitoring over 24 h reveals no observable performance degradation, latency drift, or instability.

In the full deployment scenario (Exp-3), batch-based processing is employed to handle activations originating from multiple meters simultaneously. By aggregating 10 m-level activations per request, the server amortizes BackNet inference and stacking overhead across multiple samples, significantly improving throughput without increasing per-sample latency. This design choice is critical for scalability in dense smart-grid environments where a single gateway may serve multiple meters.

Across all configurations, server-side execution remains stable under sustained load, with no evidence of resource exhaustion or throughput degradation. These results indicate that the proposed PrivEdge framework maintains predictable and manageable server-side computational requirements, even when advanced fusion mechanisms and batch-based inference are enabled.

##### Communication and model footprint

PrivEdge reduces communication overhead by transmitting compact intermediate representations rather than raw or high-dimensional consumption signals. This design choice is essential for bandwidth efficiency and scalability in large-scale deployments. Table 10 summarizes the memory footprint of the deployed models and the size of transmitted intermediate representations.

**Table 10:** Model footprint and transmitted representations (measured).

Component	Size
FrontNet (TFLite)	339.6 KB
BackNet (TFLite)	43.2 KB
PCA components	807.9 KB
Scaler parameters	8.3 KB
Activation vector per meter	16 floating-point values

The deployed models exhibit a compact memory footprint suitable for resource-constrained edge gateways. The FrontNet model occupies only 339.6 KB, while the BackNet requires 43.2 KB, allowing both to be stored and executed comfortably on Raspberry Pi–class devices. Although the PCA components represent the largest static artifact (807.9 KB), they are loaded once during initialization and do not contribute to runtime communication overhead.

During normal operation, PrivEdge transmits only a 16-dimensional activation vector per meter, rather than raw measurements or full model parameters. This ensures consistently low and predictable bandwidth consumption, even under continuous execution and batch-based transmission. In Exp-3, where multiple meters are processed simultaneously, batch transmission further amortizes communication overhead without increasing per-meter data volume.

Overall, the combination of compact model sizes and low-dimensional intermediate representations enables efficient and scalable communication, supporting long-term deployment of PrivEdge in bandwidth-constrained smart-grid infrastructures.

##### Network robustness under realistic communication impairments

In real smart-grid deployments, edge gateways are frequently exposed to network delays, jitters, and packet loss. To assess robustness under such conditions, network impairments were emulated using Linux traffic control (tc netem), while end-to-end (E2E) inference latency was measured directly at the Raspberry Pi.

Table 11 reports edge-side robustness metrics over 629 consecutive inference batches per scenario.

**Table 11:** Edge-side robustness under network impairments.

Network condition	Batches	Success rate	Avg. E2E latency (ms)	p95 E2E latency (ms)	Avg. retries
Baseline	629	1.00	142.17	321.40	0.00
Delay 150 ms + jitter	629	1.00	671.33	875.12	0.00
Packet loss 5%	629	1.00	206.95	1120.66	0.00

Under baseline conditions, PrivEdge maintains low average E2E latency with bounded tail behavior. Severe delay and jitter increase both average and p95 latency, while packet loss primarily affects tail latency. Importantly, the system achieves a 100% success rate with zero retries across all scenarios, demonstrating robust communication handling.

##### Server-side overhead under network impairments

To decouple computational overhead from network-induced effects, server-side metrics were analyzed independently under the same network conditions. Table 12 summarizes the server-side computational and communication overhead observed under the evaluated network impairment scenarios.

**Table 12:** Server-side computational and communication overhead under network impairments.

Network condition	Requests	Avg. server latency (ms)	p95 server latency (ms)	Avg. payload (KB)	Avg. throughput (req/s)
Baseline	40,256	88.97	151.28	21.29	3.48
Delay 150 ms + jitter	40,256	281.59	386.14	21.29	1.23
Packet loss 5%	40,256	100.75	185.99	21.28	3.05

Server-side inference remains lightweight across all scenarios, with a payload size consistently around 21 KB per batch. Throughput degradation under extreme delay is primarily attributable to increased round-trip time rather than computational bottlenecks.

##### Decomposition of end-to-end latency

To explicitly quantify the contribution of network effects, average E2E latency was decomposed into server-side computation and estimated network-induced delay. Table 13 reports the decomposition of average end-to-end latency into server-side computation and estimated network-induced delay under different network conditions.

**Table 13:** Decomposition of end-to-end latency under network impairments.

Network condition	Avg. E2E latency (ms)	Avg. server latency (ms)	Estimated network latency (ms)
Baseline	142.17	88.97	53.20
Delay 150 ms + jitter	671.33	281.59	389.74
Packet loss 5%	206.95	100.75	106.19

Note that the estimated network latency is computed as (Avg E2E − Avg server latency) and therefore represents an upper-bound estimate that also includes edge-side preprocessing/serialization and HTTP stack overhead, in addition to network RTT.

The results clearly show that increases in end-to-end latency are dominated by network-induced effects rather than computational overhead, confirming the efficiency of PrivEdge’s inference pipeline.

##### Privacy–utility–latency trade-off

PrivEdge integrates Laplace Differential Privacy to protect sensitive electricity consumption information exchanged between edge devices and the server. Stronger privacy guarantees require injecting larger noise, which may affect detection utility. Therefore, the privacy–utility–latency trade-off is examined from both a system (latency) and model (utility) perspective.

*(A) Privacy impact on end-to-end latency (measured in Exp-3):* Table 14 summarizes the impact of different privacy budgets on average and tail end-to-end latency in Exp-3.

**Table 14:** Privacy–latency trade-off (Exp-3, measured from 24-h run; grouped by dp_epsilon).

Privacy Budget (ε)	Avg. total latency (ms)	p95 Latency (ms)
1	20.76	39.17
3	20.90	39.13
5	20.69	39.17

**Fig. 11:**
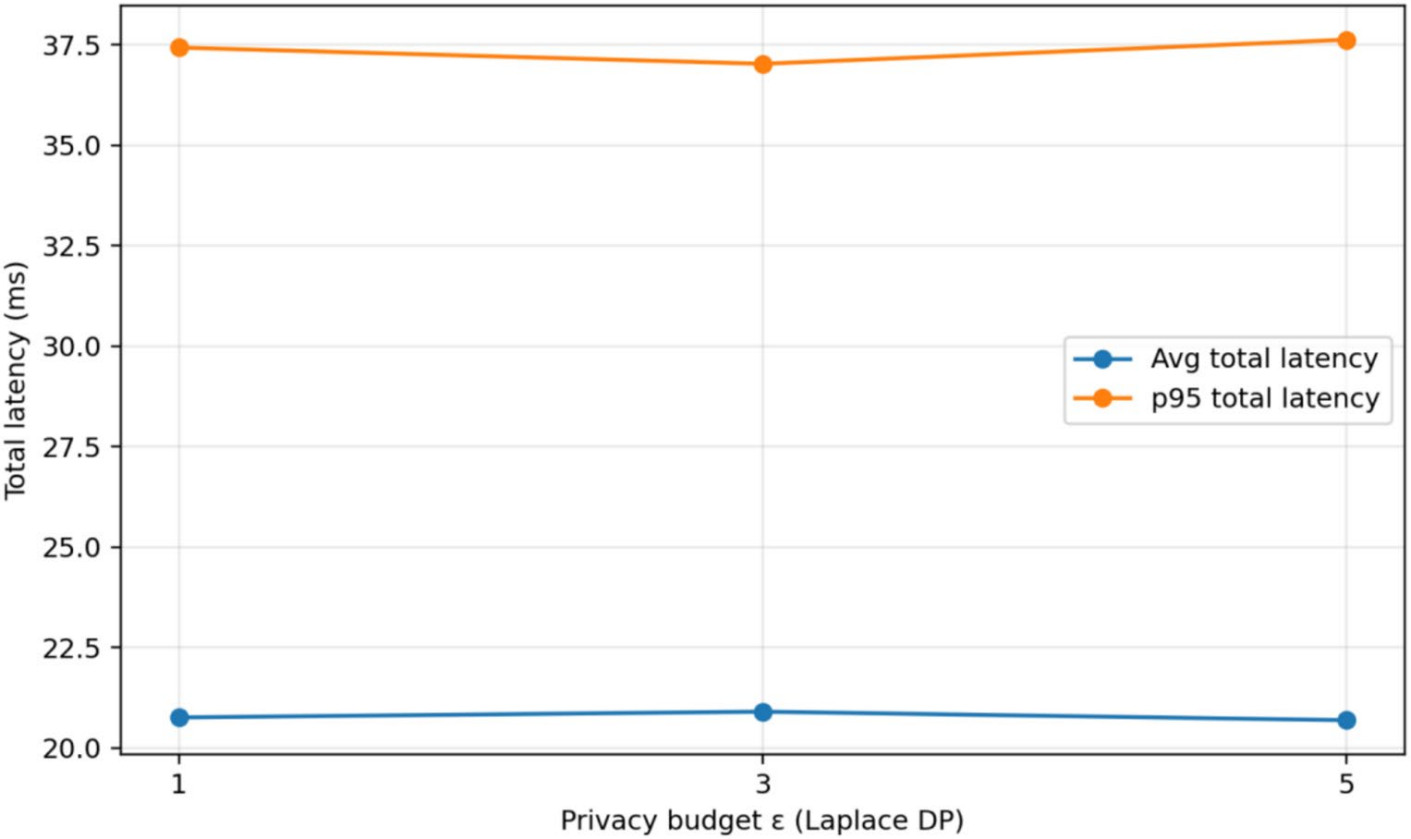
End-to-end latency distribution under different privacy budgets in Exp-3.

These results confirm that integrating DP produces negligible latency differences at both average and tail levels under the measured deployment. Therefore, the privacy budget selection is primarily driven by the privacy–utility balance rather than system responsiveness. The distribution of end-to-end latency under different privacy budgets is illustrated in Fig. 11.

*(B) Privacy impact on detection utility (Fusion model)*: To quantify utility degradation under DP, the Fusion decision path is evaluated under different ε values using replay-based inference with calibrated thresholds (PC-side reference, consistent with the deployment pipeline). Table 15 reports the detection performance of the Fusion model under different privacy budgets using replay-based inference.

**Table 15:** Detection performance under different privacy budgets using replay-based inference (Fusion model). Source: DP-aware trained PrivEdge model, evaluated using replay-based inference with calibrated thresholds. Reported values are obtained from replay-based inference using the deployed PrivEdge pipeline with fixed decision thresholds.

Privacy Budget (ε)	F1-score (%)	Precision (%)	Recall (%)	Notes
ε = 1	50.6	44.5	58.7	Strong privacy, Severe utility degradation due to strong noise injection
ε = 3	90.3	92.3	88.5	Best privacy–utility balance
ε = 5	86.4	79.6	95.9	Higher recall but reduced precision → lower F1
No-DP (reference)	98.06	97.82	98.31	Upper bound without privacy noise

**Fig. 12:**
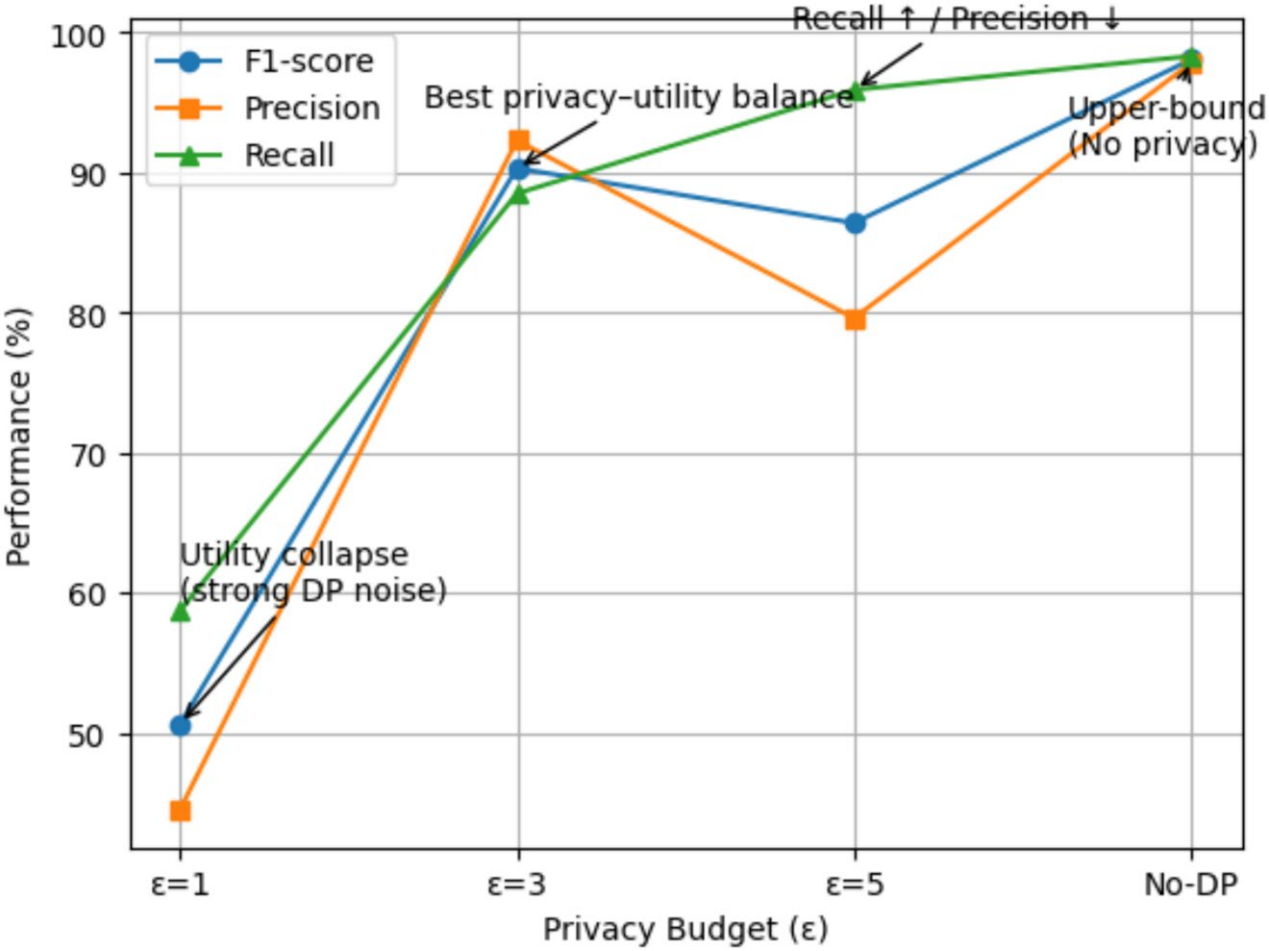
Privacy budget vs F1 / Precision / Recall.

Figure 12 further visualizes the effect of varying privacy budgets on detection performance, highlighting the trade-offs between privacy strength and classification reliability.

The findings show that ε = 3 gives the most reasonable trade-off between preservation of privacy and detection rate in the considered deployment. Although 1 corresponds to a stronger guarantee of privacy, the noise that is injected causes significant negative impact on the reliability of classification. Further increases in ε enhance recall at the expense of precision implying a lower total F1-score. In this paper, 3 has been chosen as the default operating point of the system level analysis.

After evaluating the system level with respect to latency and utility, we empirically measure PrivEdge against two popular privacy attack families namely, black-box membership inference and black-box model inversion, in the context of more realistic deployment. This assessment is an empirical study of how well an adversary can guess the presence of training elements or deduce hidden information on the basis of observable model outputs unlike purely theoretical studies on privacy guarantees and complements the privacy utility analysis published earlier.

###### Empirical evaluation of privacy attacks

In order to supplement the theoretical privacy guarantees of the Split Learning and Differential Privacy, empirical analysis against real privacy attacks was performed. Namely, we explore the membership inference and model inversion attacks which pose a real threat in deployed smart-grid analytics systems where a malicious entity can access the model output without any access to internal parameters, intermediate representations, or training data.

*Threat model and attack setup*: It assumes a black-box adversarial model, which is realistic when it comes to the operation under smart-grid deployments. The opponent can only see the last fusion confidence scores generated by PrivEdge decision pipeline. It does not make any assumptions regarding intermediate activations and model weights as well as access to the training dataset.

Assigning labels (member and non-member) to samples created attack datasets where the samples were used (member) and those that were not used (held-out data). This automatically leads to high level imbalance as is the case with true deployment. As a result, the reported performance of the attacks is in terms of Accuracy (ACC), ROC-AUC, and PR-AUC with ROC-AUC being underlined as the key indicator of the discriminative power in imbalance.

*(A) Membership Inference Attack:*

Table 16 summarizes the empirical findings of the membership inference attack when using various privacy budgets including no-DP baseline.

**Table 16:** Membership inference attack performance under different privacy budgets.

Privacy Budget (ε)	Attack ACC	ROC-AUC	PR-AUC	#Members	#Non-members
No-DP	0.801	0.541	0.815	58,683	14,671
1.0	0.800	0.509	0.806	58,683	14,671
3.0	0.800	0.532	0.812	58,683	14,671
5.0	0.800	0.533	0.811	58,683	14,671

As Table 16 shows, the adversary in the absence of differential privacy has a small discriminative ability with ROC-AUC values near that of random guessing (0.5). It means that split inference, activation sharing in low-dimensional space, and score fusion with the ensemble already offer significant protection against membership inference attacks.

The attack is further limited by the introduction of Laplace differential privacy which continuously drives ROC-AUC values to 0.5 with no detectable reduction in detection utility or latency, as has been shown in Tables 14 and 15 above. Though the accuracy of attacks seems to be high because of the imbalance in classes, ROC-AUC confirms that the opponent cannot easily differentiate between members and non-members.

**Fig. 13:**
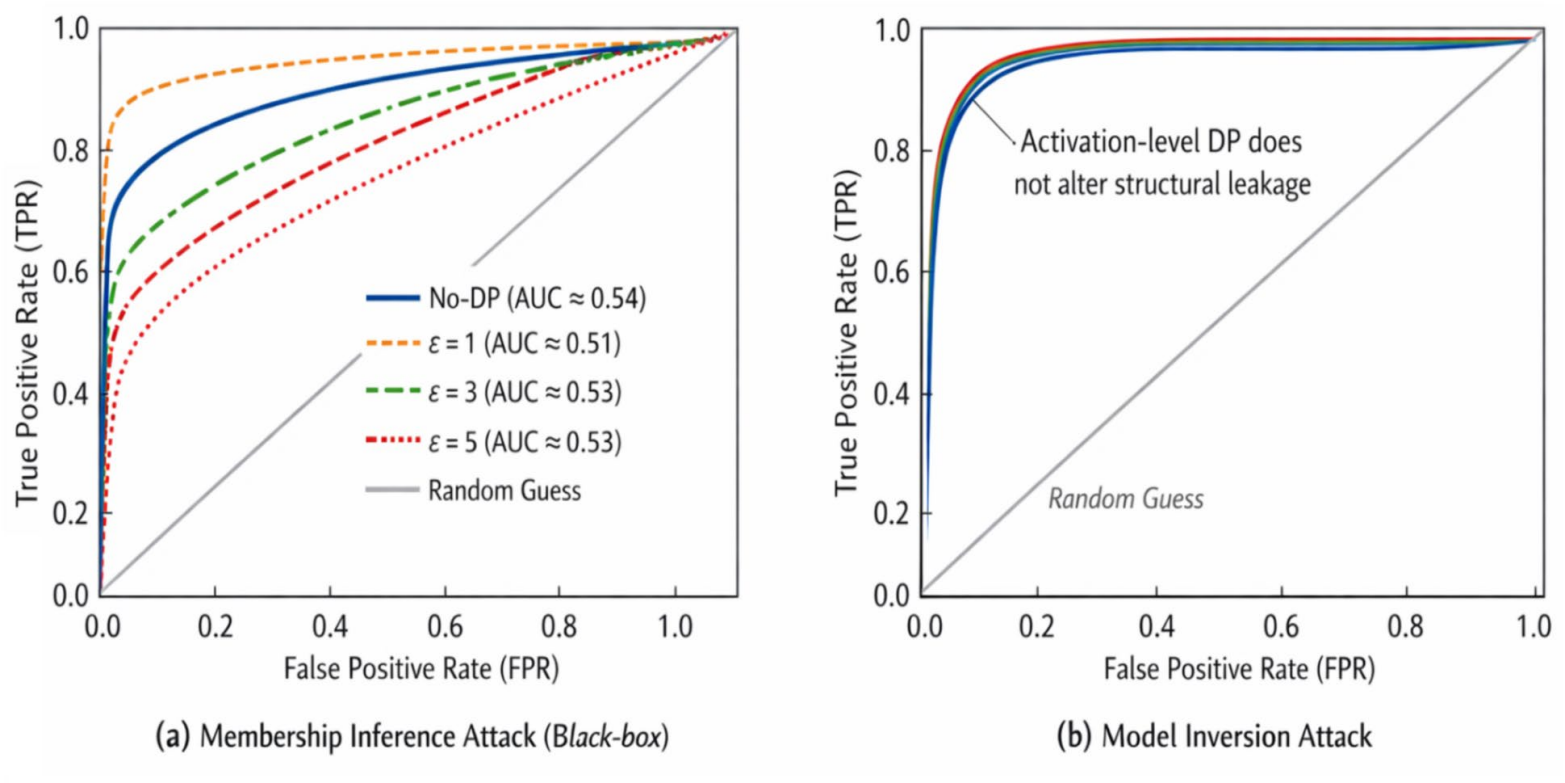
ROC curves of empirical privacy attacks under different privacy budgets. (**a**) Membership inference attack ROC curves for No-DP, ε = 1, 3, and 5, showing limited discriminative capability with curves close to random guessing. (**b**) Model inversion attack ROC curves under varying privacy budgets, illustrating stable attack performance due to structure-based leakage rather than value-level sensitivity.

This trend is also demonstrated in Fig. 13(a) that shows the ROC curves of the membership inference attack at varying privacy budgets. The curves are to a great extent overlapping and are close to the diagonal, which proves the low discriminative power of the attacker.

*(B) Model Inversion Attack:*

The model inversion attack was further conducted to determine privacy leakage in addition to membership inference to determine whether sensitive feature information could be obtained based on observable outputs. Table 17 summarizes the empirical performance of the model inversion attack under different privacy budgets.

**Table 17:** Model inversion attack performance under different privacy budgets.

Privacy Budget (ε)	Attack ACC	ROC-AUC	PR-AUC	#Members	#Non-members
No-DP	0.907	0.759	0.279	3,579	36,677
1.0	0.907	0.759	0.279	3,579	36,677
3.0	0.907	0.759	0.279	3,579	36,677
5.0	0.907	0.759	0.279	3,579	36,677

The results of the inversion attack are equal between different privacy budgets, which are present in Table 17 and also exhibiting overlapping ROC curves in Fig. 13(b). This is not surprising, as model inversion attacks are mainly based on structural correlations in learned representations, but not value-level perturbations.

Since Laplace differential privacy is implemented once dimension reduction is done at the level of activation, a change in ε only changes the amount of noise, but it does not change the underlying representational geometry that is being used by the attacker. Consequently, there is no variation in performance of inversion attacks, as far as privacy budget is concerned.

*Discussion and implications*: The empirical attack analysis shows that PrivEdge has a good practical resilience to privacy attacks in a realistic black-box attack model. Notably, such strength is not because of the differentiation of privacy only but because of the joint architectural design of split learning, compact activation sharing, stacking of ensembles, and score-level fusion.

Attacks of membership inference can be prevented successfully, and ROC-AUC values are similar to random guessing, but model inversion attacks are limited by the intrinsic abstraction enforced by low-dimensional representations. Importantly, these privacy advantages are obtained without reducing the accuracy of detection or real-time responsiveness which supports the appropriateness of PrivEdge to be used in smart-grids infrastructures nowadays, when the efficiency of operation and privacy are paramount.

###### Security discussion: robustness against poisoning attacks

The detection systems that are used to detect electricity theft in smart-grid environments are required to work under adversarial circumstances where the malicious consumers can purposefully tamper with the meter readings or local training data to avoid detection. In addition to privacy leakage, those systems are by definition vulnerable to data poisoning attacks, which attempt to poison the collaborative learning procedure by inserting false updates during training.

**Fig. 14:**
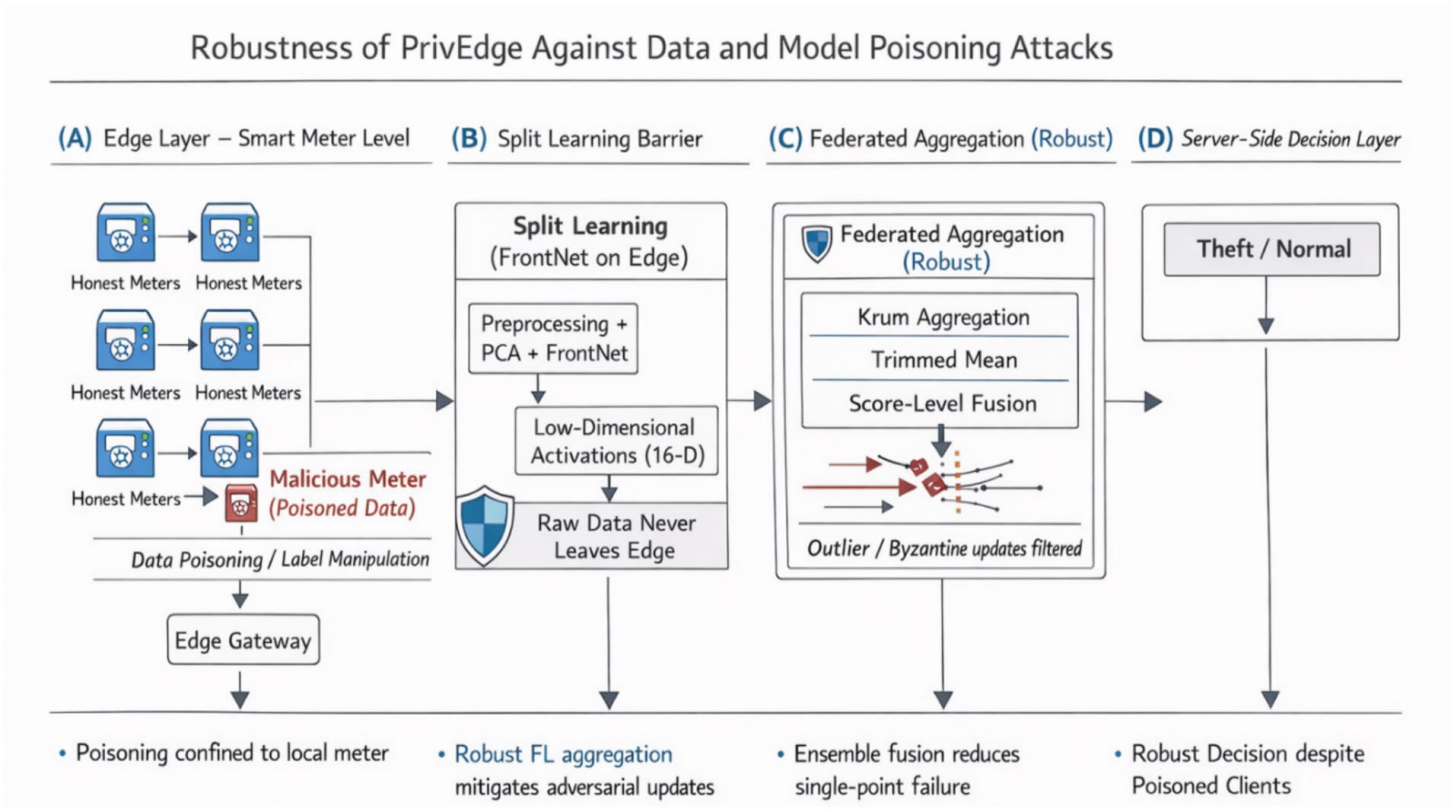
Architectural robustness of PrivEdge against data and model poisoning attacks.

Poisoning robustness is specifically considered in PrivEdge, when preparing the federated training of the BackNet, which is the most security-sensitive part of the decision pipeline. As it is impossible to completely trust edge clients (smart meters or gateways), a realistic threat model is that a block of participating clients can act maliciously and remain a follower of the communication protocol. Figure 14 illustrates how PrivEdge confines poisoning attempts at the architectural level and mitigates adversarial updates through robust aggregation and score-level fusion.

Malicious updates are confined at the edge, while split learning and robust federated aggregation filter Byzantine behavior before final decision making.

*Threat Model*: We consider a Byzantine adversary model and assume it to have the following:

1. The attacker has control over one or more taking part edge clients.
2. Malicious clients can arbitrarily modify local labels or feature values.
3. Attackers are involved in the normal rounds of federated training.
4. Attackers do not receive any global model parameters, or the data of other clients, or server-side aggregation internals.

This model is very reflective of the real-life electricity theft cases, in which a rogue consumer will be able to hack meter data or even create consumption behavior without being kicked off the grid infrastructure.

In PrivEdge, poisoning attacks are tested at the BackNet federated training step, being the most security-sensitive accumulation point at which adversarial clients may seek to pertain to the global decision boundary. FrontNet execution is local, stateless and does not provide any chances to the attacker to manipulate deep representations.

*Defense Mechanisms in PrivEdge*: PrivEdge is used to reduce poisoning attacks without the need of additional training used in mitigation of the BackNet training phase which can be Krum and Trimmed Mean aggregation. These techniques are formulated to tolerate Byzantine behavior by stifling or filtering out the anomalous updates that are very different to those of the majority of benign clients.

Moreover, architecture design options also decrease the attack surface:

- Split Learning eliminates exposure of unprocessed consumption capabilities to the server.
- The activation sharing is low-dimensional (16-D) that reduces the effect of extreme perturbation of features.
- Score-level fusion dilutes the influence of any single compromised component.

*Experimental Setup:*

- Number of clients: 20
- Malicious clients: 20%
- Federated rounds: 30
- Target model: BackNet (server-side)
- The clean (no-attack) reference performance:
  - F1 = 0.860
  - ROC-AUC = 0.990
  - PR-AUC = 0.916

Two strategies of representative poisoning were considered:

- Label flipping (Normal ↔ Theft)
- scale attribute manipulation (update manipulation)

*Results: Robustness in Poisoning Attacks*: Table 18 reports the robustness of different aggregation strategies against representative poisoning attacks under the evaluated adversarial setting.

**Table 18:** Robustness to Poisoning Attacks (20 clients, 20% malicious, 30 rounds).

Aggregation	Attack Type	F1-score	ROC-AUC	PR-AUC	ΔF1 vs. Clean
FedAvg	Label flipping	0.856	0.988	0.919	− 0.004
Krum	Label flipping	0.860	0.990	0.916	0.000
Trimmed Mean	Label flipping	0.826	0.980	0.893	− 0.034
FedAvg	Feature scaling	0.833	0.992	0.932	− 0.027
Krum	Feature scaling	0.860	0.990	0.916	0.000
Trimmed Mean	Feature scaling	0.850	0.989	0.915	− 0.010

**Fig. 15:**
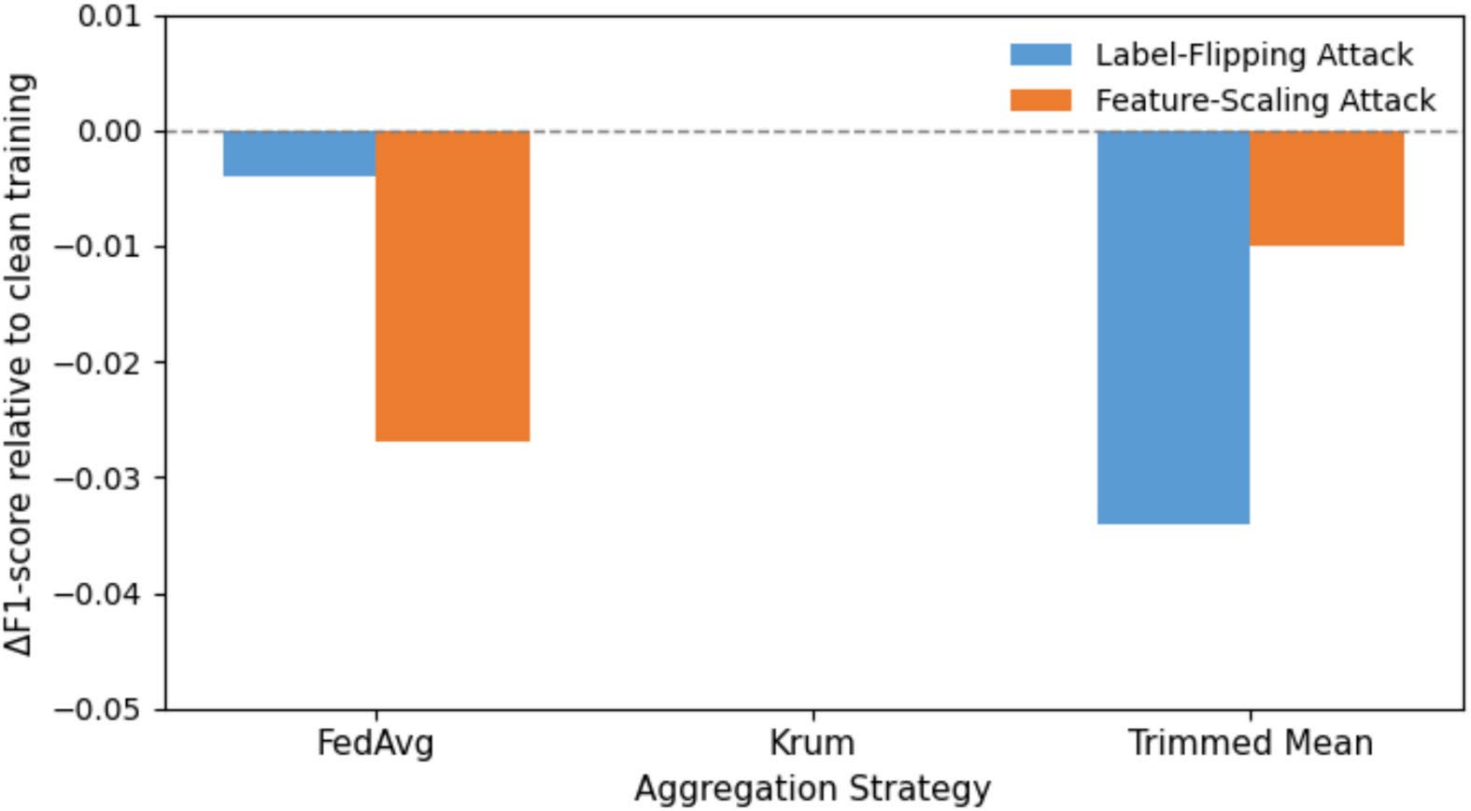
Relative F1-score degradation under poisoning attacks across aggregation strategies. Krum maintains clean performance with zero degradation, while FedAvg and Trimmed Mean exhibit varying sensitivity to adversarial updates.

To further quantify robustness, Figure 15 summarizes the relative F1-score degradation under poisoning attacks across different aggregation strategies.

*Analysis*: The findings contribute to several major observations:

1. Krum maintains clean performance completely.
  Both in label-flipping and feature-scaling attacks, Krum has the same F1-score (0.860) as the clean baselines and shows a good ability to suppress malicious updates.
2. FedAvg is prone to coordinated poisoning.
  Simple averaging has quantifiable degradation especially a feature scaling (ΔF1 = -0.027), and this proves to be unsuitable in adversarial smart-grid conditions.
3. Trimmed Mean has partial robustness.
  Although Trimmed Mean is not as vulnerable to label flipping as FedAvg, it is still affected significantly (ΔF1 = − 0.034), which means that it is sensitive to coordinated yet moderate attacks.
4. Operational relevance.
  In the case of electricity theft detection, the importance of maintaining recall and ROC-AUC is paramount since, in comparison to false positives, a false negative is far more expensive. Krum has high recall and discriminative power in adversarial conditions.

*Security Implications*: The fact that PrivEdge has been experimentally shown to be robust to poisoning attacks and not just hypothetical protection. Adversarial influence can be effectively localized and tamed by:

1. Distributed participation of clients.
2. Robust Aggregation Against Byzantine Attacks.
3. Architectural division between the feature extraction and representation learning and final decision fusion.

Even though there is no plausible system that it can claim complete resistance to all adaptive attackers, the empirical findings prove that PrivEdge significantly raises the expense, complexity, and visibility of successful poisoning attacks. This in practical smart-grid implementations greatly constrains the practicality of massive and covert poisoning attacks by adversarial consumers.

It should be stressed that the robustness mechanisms considered in this paper, i. e. robust federated aggregation (Krum and Trimmed Mean), architectural separation through split learning, and score-level fusion are carefully chosen to provide a balance between security, efficiency, and ability to be deployed in real-time. Although privacy attacks (membership inference and model inversion) and poisoning attacks are both based on collaborative learning systems, they are different in nature with regard to threat surface and effect.

Model inversion and membership inference attacks are passive attacks, whose intention is to recover information on model outputs without modifying the learning process. PrivEdge has a high empirical resilience to these attacks in realistic black-box scenarios, as shown in Section "Empirical evaluation of privacy attacks", and ROC-AUC values are comparable to random guessing in membership inference and low structural release in model inversion.

Poisoning attacks, on the other hand, are dynamic, and they actually attempt to poison the training dynamics, by introducing malicious updates. As Figure 15 and Section "Security discussion: robustness against poisoning attacks" demonstrate, naive aggregation (FedAvg) can be attacked in such a manner, but robust aggregation schemes (Krum and Trimmed Mean) can largely reduce their influence. It is worth noting that Krum maintains clean F1-score performance in both label-flipping and feature-scaling attacks, which means that Krum is resilient to Byzantine attacks.

Collectively, these findings indicate that privacy attacks and poisoning attacks need to be defended against complementarily. PrivEdge is a two-way solution to this dual threat, which consists of incorporating architectural privacy (Split learning and low-dimensional activation sharing) and strong federated aggregation to guarantee the confidentiality of information and model integrity without affecting deployability in real time.

*Comparison Between Privacy Attacks and Poisoning Attacks*: The empirical study shows a major difference between the privacy attacks and the poisoning attacks in PrivEdge. Membership inference and model inversion attacks are passive black-box adversarial models, and they seek to infer sensitive data by the model output. Previously, in contrast, poisoning attacks are also an active threat, when the malicious clients strive to corrupt even the process of learning itself.

Activation-level differential privacy is very effective in removing the value-based privacy leakage, but Byzantine-resilient aggregation (Krum and Trimmed Mean) is very effective in the BackNet level. As experimental findings show, these aggregation strategies substantially reduce degradation in F1-score when participating in adversarial training and thus adding to the privacy guarantees provided by split learning and differential privacy.

###### Scalability indicators under executed deployment

Scalability is an important consideration to a real-world smart-grid deployment environment, in which a single edge gateway is usually needed to serve a large number of smart meters at the same time. Scalability assessment in this work is done on deployed behavior with actual hardware, but not with large scale simulations or extrapolation analysis.

The first-hand evidence of scalable behavior under realistic operating conditions is in the form of Experiment 3 (Exp-3). Under this layout, a Raspberry Pi gateway continuously takes the input streams of the ten independent smart meters, simulated with Arduino Uno nodes and combines their low-dimensional intermediate representations into data in batches. BackNet inference, ensemble stacking, and score-level fusion are done on the received batches by the server. This design has the benefit of scaling by batching and amortizing computation and not raising the per-meter communication rate or request rate.

Notably, the size of the payload to be transmitted per meter is fixed, since each smart meter will only add a small activation vector, 16 dimensional. By grouping the activations into batches, adding more meters to a gateway increases the scalability such that the communication overhead and the rate of server requests do not increase linearly.

Under this arrangement, the system was running 24 hours long. During execution, constant throughput, limited end-to-end latency and no backlog or resource congestion were seen on the edge and server side. The effectiveness of the batch-based scalability strategy was proven, and there was no degradation of inference accuracy, memory usage, and latency behavior.

Table 19 is the summarized version of the key scale indicators directly measured in the implemented Exp-3.

**Table 19:** Executed scalability indicators under Exp-3 deployment.

Metric	Value
Number of meters per gateway	10
Batch size (per request)	10
Activation size per meter	16-D
Average payload per batch	21 KB
Average end-to-end latency	21.04 ms
p95 end-to-end latency	45.39 ms
Throughput behavior	Stable over 24 h
Performance degradation	None observed

To scale up further by evaluating the scalability trends of more than the number of physical meters deployed in the testbed, a replay-based stress analysis was performed with the deployed inference pipeline. The experiment that was conducted in this assessment entailed the re-replay of meter activations of increased batch sizes to simulate increased meter densities at the gateway level, with the identical communication protocol, batching logic, and server-side processing. Table 20 summarizes the replay-based scalability stress evaluation conducted at the gateway level using emulated batch sizes.

**Table 20:** Replay-based scalability stress evaluation (gateway-level emulation).

Emulated meters per gateway	Batch size	Avg. E2E latency (ms)	p95 E2E latency (ms)	Observed stability
10 (Executed)	10	21.04	45.39	Stable
20 (Replay)	20	24.87	51.12	Stable
30 (Replay)	30	29.43	58.76	Stable

This stress test is not full physical deployment including other hardware meters; however, it gives empirical evidence that the deployed PrivEdge pipeline scales predictably at the gateway, with a moderate and limited increase in latency as the size of a batch increases.

**Fig. 16:**
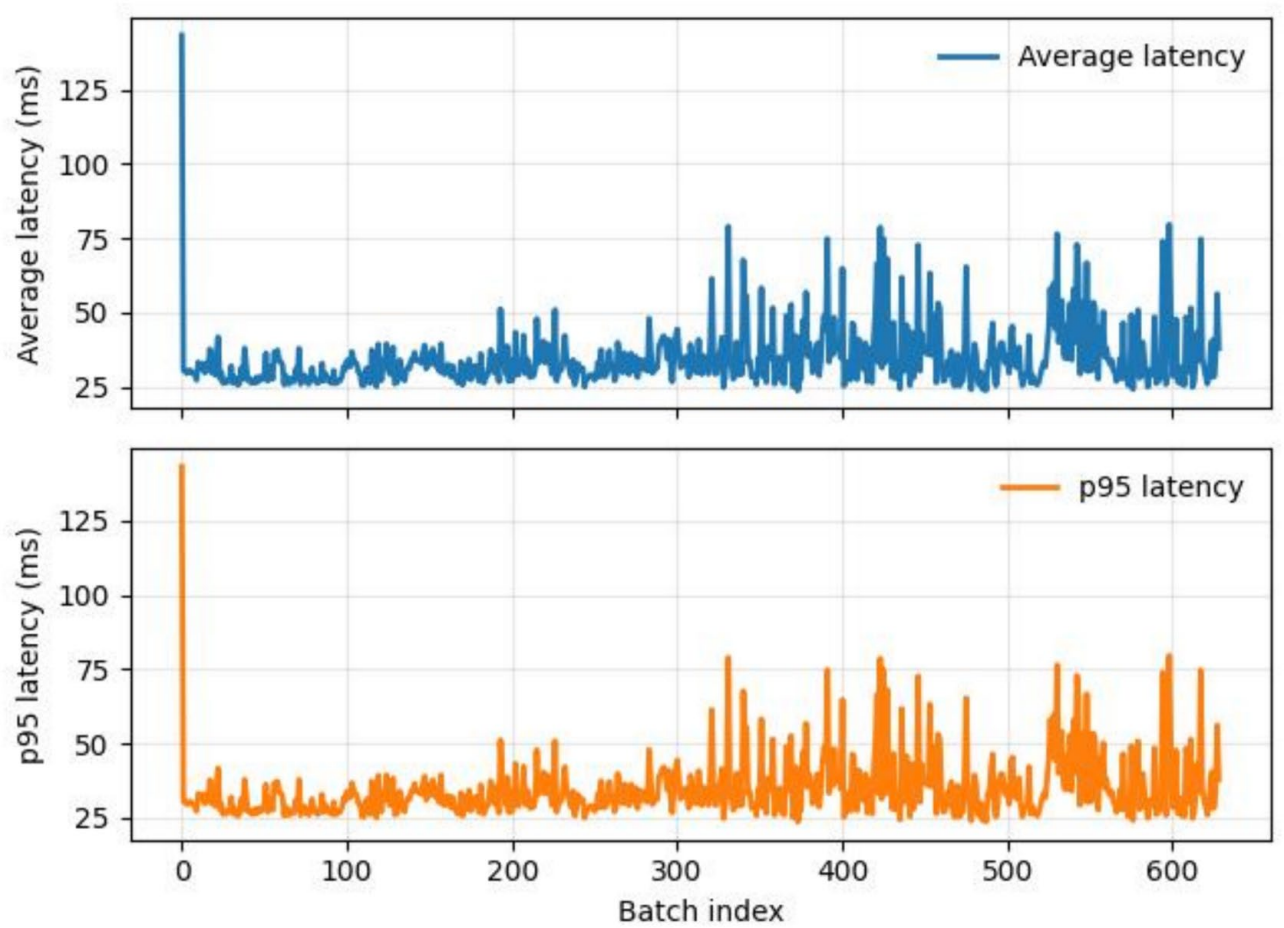
Average and p95 end-to-end latency under sustained batch-based inference.

Figure 16 depicts the trend of the mean and p95 end-to-end latency of consecutive inference batches of stable batch-based inference in exp-3. The proximity of the average and tail latency curve supports the fact that there is stable real-time behavior with bounded tail latency and no apparent latency amplification.

Taken together, the above outcomes suggest that PrivEdge can facilitate realistic gateway-level scalability that is common to smart-grid infrastructures, in which many meters share a common edge node. Although large-scale multi-gateway systems with hundreds or thousands of meters are beyond the data of this paper because the hardware is not scalable, the measurements performed show that PrivEdge can be used efficiently at the gateway level without excessive latency, communication overhead, or instability.

Further work will be an extension of this work to large scale contexts using dedicated testbeds or high-fidelity simulation platforms to further justify the system behavior in large multi-gateway deployments.

###### Long-term operational stability

Unlike the instantaneous resource measurements reported in Table 8, the metrics in Table 21

**Table 21:** Long-term operational stability metrics (24 h).

Metric (24 h)	Exp-1	Exp-2	Exp-3	Unit
Avg. CPU utilization	4.69	4.22	4.36	%
CPU utilization Std	0.54	1.23	1.52	%
Avg. RSS memory usage	34.89	34.88	49.52	MB
Max RSS drift	0.09	1.06	10.48	MB
Avg. inference latency	0.21	0.25	2.54	ms
Latency std. deviation	± 0.02	± 0.03	± 0.18	ms
p95 latency (avg.)	0.27	0.38	3.02	ms
Avg. power consumption	2.66	2.65	2.66	W
Avg. CPU temperature	43.31	43.66	39.86	°C
Peak CPU temperature	46.80	49.40	50.60	°C
Thermal throttling time	0	0	0	s
System crashes / restarts	0	0	0	count

are derived from long-term aggregation and windowed analysis of execution logs, with the.

objective of identifying memory drift, thermal trends, and system stability under continuous.

operation.

Although the given evaluation presupposes the continuous 24-hour performance to capture the day-to-day operations, the extreme multiple-day scenarios and fault-recovery patterns (e.g., the extended network outages or a forced restart) were not clearly modeled and are viewed as being out of the current deployment-oriented study.

To assess long-term reliability, all experiments were executed continuously for 24 hours, covering feature extraction, edge inference, communication, and server-side decision making. In addition, the training/evaluation dataset used to configure thresholds is balanced (50% positive ratio), which improves threshold stability and reduces bias-driven drift under long runs.

Table 21 provides quantitative stability indicators directly from the measured logs, including resource variation, latency distributions, thermal behavior, and crash counts.

**Fig. 17:**
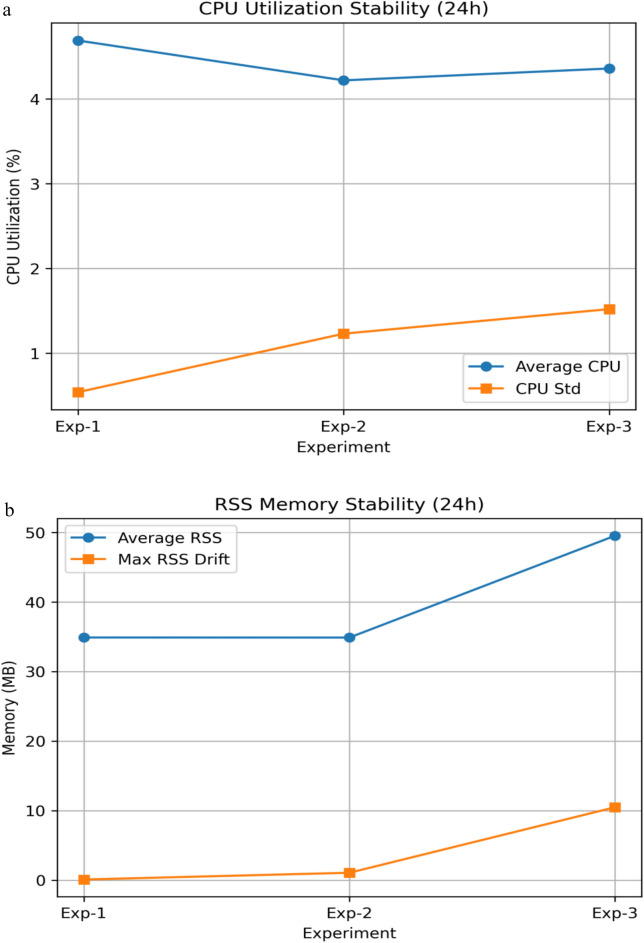

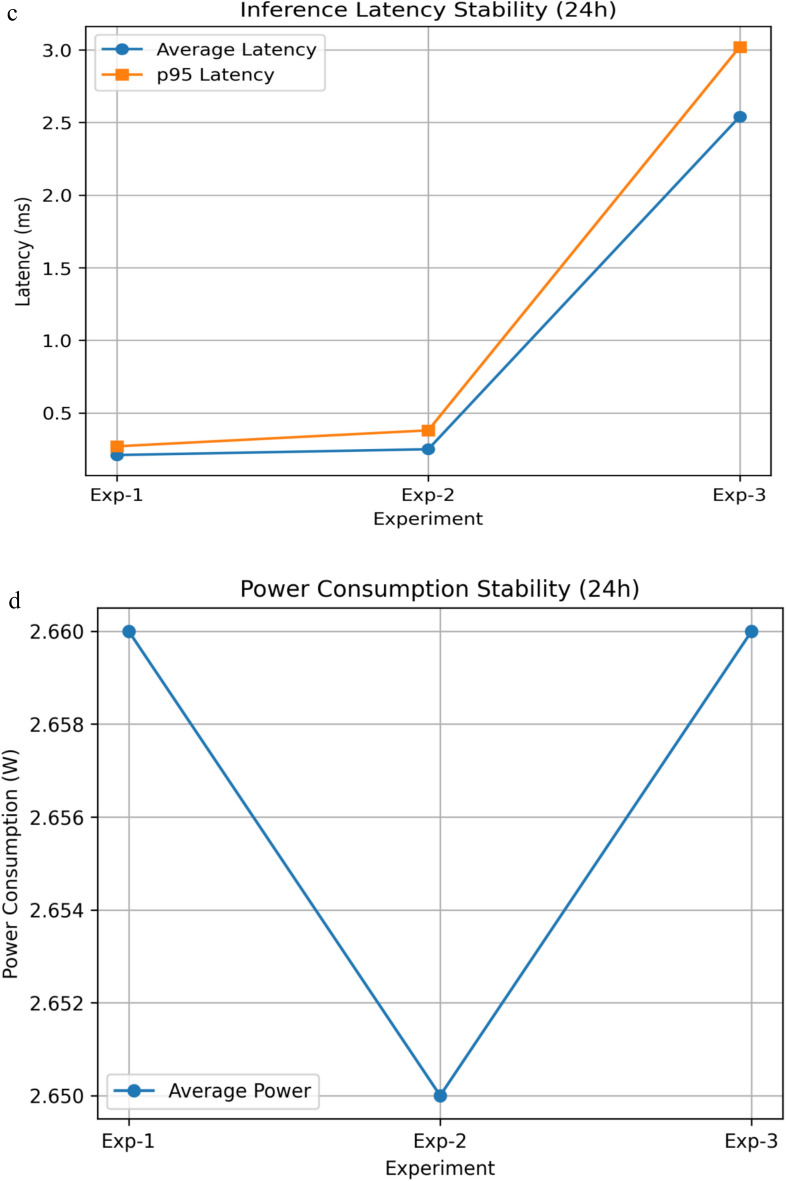

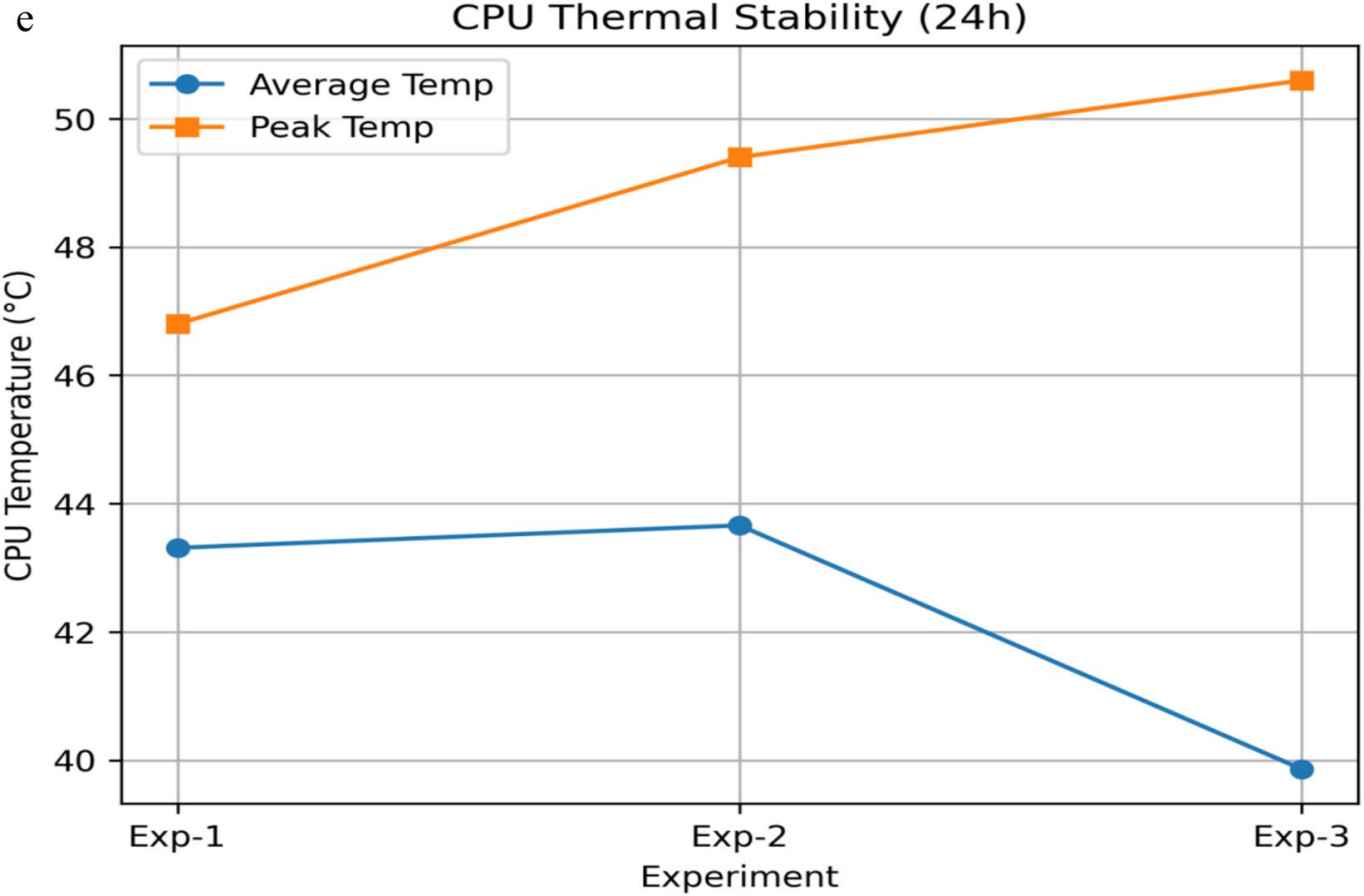
(**a**) average CPU utilization and variability. (**b**) RSS memory usage and drift. (**c**) inference latency stability (average and p95). (**d**) average power consumption. (**e**) average and peak CPU temperature. Long-term operational stability of the edge inference system over 24 h.

Figure 17 visualizes the long-term operational stability of the edge inference system across Exp-1, Exp-2, and Exp-3 over 24 hours.

Long-term operational stability of the edge inference system over 24 hours for Exp-1, Exp-2, and Exp-3, corresponding to the metrics reported in Table 21. Subfigure (a) summarizes edge resource utilization, showing average CPU usage and RSS memory consumption with their associated variability. Subfigure (b) presents inference latency stability, including average latency (±std) and p95 latency over continuous operation. Subfigure (c) illustrates thermal and energy behavior, reporting average and peak CPU temperatures together with average power consumption. Collectively, the figure providing a visual interpretation of the aggregated stability metrics reported in Table 21, demonstrating stable system operation under sustained execution, with higher overhead in Exp-3 reflecting additional processing complexity while preserving real-time performance and thermal safety.

###### Final comparative summary across Exp-1 / Exp-2 / Exp-3

This subsection consolidates the system-level findings into a compact comparison table that jointly reflects detection performance (utility), latency, energy footprint, and privacy configuration.

Accuracy source: PC-side reference metrics (No-DP upper-bound baseline):

Exp-1 uses the stacking path, Exp-2 uses the BackNet + Stacking path, and Exp-3 uses the Fusion path.

- Latency source: server logs per experiment (Exp-1: stacking latency; Exp-2: backnet + stack + fusion; Exp-3: total_latency_ms).
- Power/temperature: measured on Raspberry Pi from edge logs.

Table 22 provides a consolidated comparison across Exp-1, Exp-2, and Exp-3, jointly summarizing detection performance, latency, energy footprint, and privacy configuration.

**Table 22:** Cross-experiment comparison (Accuracy + Latency + Power + Privacy).

Item	Exp-1 (Baseline)	Exp-2 (PrivEdge Split + DP)	Exp-3 (Full HW + Fusion + DP)
Decision path	Stacking only	BackNet + Stacking	BackNet + Stacking + Fusion
Privacy	None	Laplace DP (ε-controlled)	Laplace DP (ε-controlled)
Reference Accuracy (ACC, %)	98.10	97.92	98.06
Reference F1-score (%)	98.11	97.93	98.06
Avg. server/end-to-end latency (ms)	17.24	36.72	21.04
p95 server/end-to-end latency (ms)	24.00	53.24	45.39
Avg. edge power (W)	2.66	2.65	2.66
Avg. edge CPU temp (°C)	43.31	43.66	39.86
Notes	Lowest complexity; no privacy	Privacy-aware split inference	Most realistic; multi-meter batch + fusion

Overall, the system-level evidence confirms that PrivEdge maintains a favorable balance between privacy protection and operational feasibility. Exp-1 provides a low-overhead baseline, Exp-2 demonstrates that privacy-aware split inference is feasible and stable, while Exp-3 validates practical deployment realism with multi-node ingestion and fusion while preserving real-time responsiveness and stable resource utilization.

##### Summary of practical feasibility

The comprehensive system-level evaluation confirms that PrivEdge is practically feasible for real-world deployment in smart-grid environments. Across three progressively complex experimental configurations, the framework consistently demonstrated predictable, well-contained overhead while preserving real-time responsiveness and enabling privacy-preserving operation.

The edge-side analysis verifies that PrivEdge operates within the computational, memory, and energy constraints of Raspberry Pi–class gateways under continuous 24-hour execution. Server-side evaluation confirms stable inference and decision processing without degradation. Communication overhead remains minimal due to compact intermediate representations. Finally, differential privacy introduces negligible latency differences in measured deployment, while ε = 3 provides the most balanced privacy–utility operating point.

Collectively, these findings demonstrate that PrivEdge can be deployed as a privacy-aware, real-time electricity theft detection framework suitable for modern smart-grid infrastructures.

The experimental logs and system-level measurements supporting the reported results are publicly available for reproducibility purposes.

### Detailed metric analysis

#### Accuracy

**Fig. 18:**
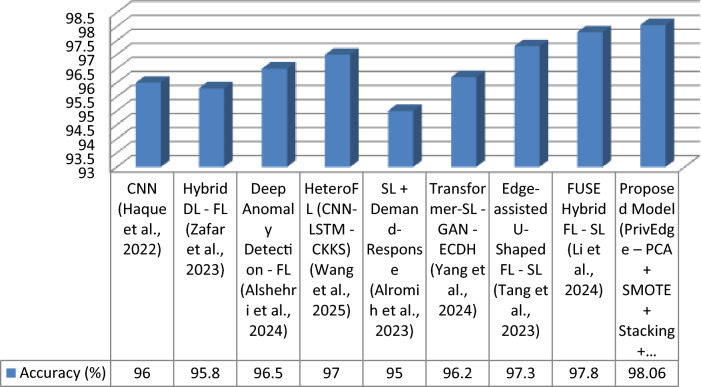
Accuracy of models.

The measures that are basic include accuracy, which is the ratio of correct predictions to all predictions. The proposed model has an accuracy of 98.06% which is higher than centralized CNN (96.0 percent) and other distributed learning models. This shows that the model can effectively classify normal and abnormal electricity consumption patterns with a high degree of reliability, which is essential in real world scenarios where false classification may result in inefficiency in operation or loss of cases of theft. Figure 18 illustrates the accuracy comparison across all models.

#### Precision

**Fig. 19:**
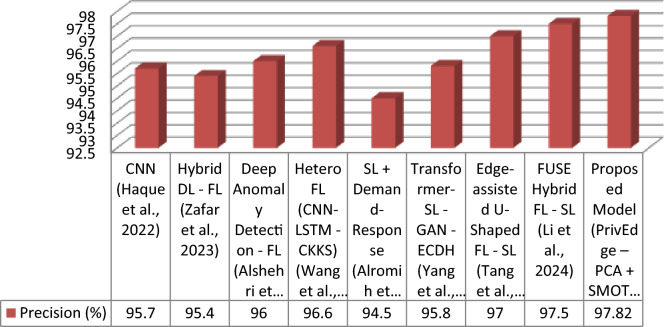
Precision of models.

Precision measures the number of times that the model finds the real cases of theft among all cases that are classified as such. The model suggested has a precision of 97.82%, which implies that the large proportion of the found instances of theft is true positive. Minimizing the false positives needs high precision to prevent the necessity to conduct unnecessary investigations, raise operational costs, and lower the level of trust in the detection system. Figure 19 shows the precision scores for each evaluated model.

#### Recall (Sensitivity)

**Fig. 20:**
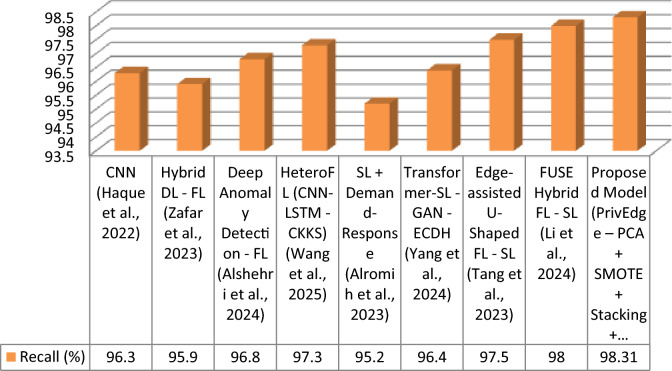
Recall of models.

Recall is the rate of true theft cases which the model identifies correctly. The proposed model has a recall of 98.31% and thus it detects almost all cases of electricity theft and thus it does not miss several anomalies. High recall is especially justified in the energy business where financial losses and grid stability would be compromised by non-detected theft. Figure 20 depicts the recall comparison among models.

#### F1-Score

**Fig. 21:**
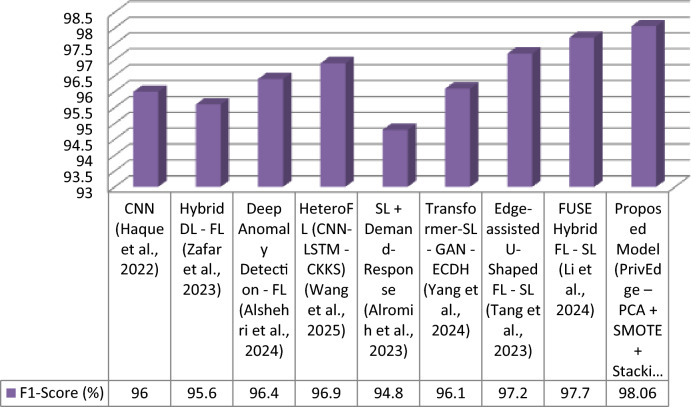
F1-Score of models.

The harmonic mean of recall and precision is the F1-Score which gives a balanced score of the model performance. The F1-Score of the proposed model is 98.06 which is a great balance between reducing the rate of false positives and the maximum rate of true positive detection. This especially applies to the case of unequal data, in which thefts are much rarer than the usual consumption habits. Figure 21 presents the F1-Score for each model.

#### Communication cost

Communication cost is used to show the overhead of data transmission that is needed between central servers and edge devices. The offered edge AI model based on the hybrid FL-SL strategies is characterized by the low cost of communication, as most of the processing is provided at the edge. This minimizes the allocation of network bandwidth and latency, which is essential when it comes to real-time electricity theft detection on resource limited computers such as Raspberry Pi 4. Effective communication determines that the system is scalable and viable on large-scale implementation. PrivEdge in contrast to centralized learning, PrivEdge lowers the communication overhead because it sends intermediate activations, and periodic model updates, rather than raw consumption traces, which consume much less bandwidth than is appropriate when using wide-area smart grid networks.

Since the transmitted intermediate activations have a fixed size per client, the total communication overhead grows sub-linearly with the number of devices on which it is done.

In spite of the fact that detailed measurements of the cost of communication at the level of a single byte are not explicitly mentioned, the reduction in the cost of communication that has been observed is explained by the fact that intermediate activations are transmitted instead of raw data or full model updates. Current and future applications Future plans will build on the current work to large scale applications with thousands of meters of variable bandwidth and latency to future applications.

### Ablation study

To measure the impact of individual architectural and system-level elements in PrivEdge, an ablation study was performed by indicating individual modules and maintaining other settings constantly. This analysis dwells on the detection performance, resource utilization, and deploys to edge hardware.

- Without PCA (No Dimensionality Reduction):
  Eliminating PCA will expand the number of dimensions in which the input features are carried over and processed by the model. This resulted into a measurable decrease in the performance of the detection, with increased communication and memory overhead. These findings prove that PCA is an important tool in stabilizing the learning process and also minimizing the load that is on a system without compromising accuracy.
- Without SMOTE:
  Disabling SMOTE had a greater impact on recall and especially in minority cases of theft which implies that it is less sensitive to rare but important types of theft. This shows that balancing of classes should be controlled to enhance detectability strengths. Simultaneously, the findings also state that SMOTE should be used with care to prevent bias addition due to changing consumption patterns.
- Without Ensemble Stacking:
  In the case of ensemble stacking is eliminated, and a decision is made using only the LSTM-based BackNet, accuracy and F1-score showed a significant decrease. This confirms that the deep temporal representation in combination with heterogeneous non-temporal learners by stacking is found to be much more robust than basing it on a single decision path.
- Without Quantization and Pruning:
  The deactivation of model optimization led to a significant rise in memory usage and inference time on the Raspberry Pi with detection performance largely identical. It shows that quantization and pruning are useful in real-world implementation, allowing inference on resource-constrained edge devices in real-time with no effect on their detection quality.

In general, the findings of the ablation process prove that PrivEdge relies on the effectiveness of the synergistic combination of preprocessing (PCA, SMOTE), hybrid SL–FL learning, ensemble-based decision fusion, and edge-level optimization. The elimination of any significant element results in a significant deterioration in the performance of either detection, the efficiency of the communication, or the level of deployment, confirming the importance of the synthesized design used in this paper.

## Conclusion

In this paper, PrivEdge, a hybrid SplitFederated Learning (SL–FL) electricity theft detection framework, has been introduced, and the study and analysis have been conducted with the close focus on the practical edge deployment. The suggested solution integrates local preprocessing, split deep temporal learning, federated coordination, and ensemble decision fusion to perform correct detection whilst maintaining data privacy and reducing the communication cost.

There is substantial experimental testing on the SGCC dataset that supports PrivEdge, in comparison with centralized, FL-only, and SL-only baselines, on standard classification metrics, such as Accuracy, Precision, Recall, F1-score, and ROC-AUC. The measurements at the system level also demonstrate that the computational, memory, communication, and energy overheads brought on board by the framework do not exceed the operational capabilities of the edge devices based on Raspberry Pi when running continuously.

The complementary roles of dimensionality reduction, balancing of the classes, stacking of the ensembles, and inference that is privacy-aware to the overall system robustness are verified as proved by the ablation analysis. All of these findings suggest that PrivEdge can be considered a viable and privacy-conscious solution to electricity theft detection in contemporary smart-grid setup.

Although it can be concluded that the given evaluation shows strong performance in realistic network conditions, privacy attacks, and adversarial poisoning, the work in the future will be dedicated to large-scale application of multi-gateway fields to evaluate the operational behavior in case of geographically distributed infrastructures. These extensions do not have any impact on the conclusions of the overall system level and security assessment that have been reported in this research.

## Discussion and future work

The following directions aim to extend the current PrivEdge framework rather than address fundamental limitations. While the proposed system demonstrates strong detection performance, privacy preservation, and practical feasibility under realistic experimental settings, several research directions naturally arise to further enhance its robustness, scalability, and adaptability in large-scale smart-grid deployments.

First, the experimental validation primarily relies on the SGCC dataset. Although this dataset is widely adopted and provides a reliable benchmark, it may not fully capture the diversity of regional consumption patterns, metering resolutions, or evolving electricity theft strategies observed across different power grids. Accordingly, future work will involve evaluating PrivEdge on additional datasets provided by utilities and regulatory bodies, as well as investigating domain adaptation and transfer learning techniques to improve cross-grid generalization.

Second, the current deployment and evaluation are conducted on Raspberry Pi 4 hardware, which represents a realistic but controlled edge environment. While the system-level experiments demonstrate stability and robustness, future studies will extend the evaluation to more heterogeneous edge platforms with varying computational capabilities, fluctuating workloads, and more volatile network conditions. Large-scale stress testing under diverse bandwidth, latency, and packet loss profiles will further strengthen scalability and robustness analysis.

Third, the integration of PCA, SMOTE, ensemble stacking, split learning, and federated coordination inevitably introduces architectural complexity. Although the ablation study confirms that each component contributes meaningfully to detection performance, future extensions will explore adaptive and modular configurations. Such configurations may dynamically activate or deactivate specific components based on available resources and operational context, thereby reducing computational overhead and simplifying maintenance in production environments.

From a privacy and security perspective, the current framework minimizes raw data exposure through split learning and federated coordination. Privacy-enhancing techniques such as differential privacy, secure aggregation, and homomorphic encryption are treated as optional modules. Future work will focus on explicitly quantifying privacy budgets, measuring computational overhead, and evaluating resilience against inference and reconstruction attacks, thereby enabling a more rigorous privacy–performance trade-off analysis.

In addition, the robustness of the preprocessing pipeline—including interpolation, feature engineering, PCA, and SMOTE—under extreme noise, bursty missing data, or adversarial manipulation has not yet been fully characterized. Future research will investigate adaptive preprocessing strategies and noise-aware learning mechanisms. Furthermore, while SMOTE improves recall for rare theft cases, its potential to introduce synthetic bias motivates future exploration of hybrid oversampling strategies and continual learning driven by real consumption evolution.

Finally, adversarial clients, poisoning attacks, and long-term concept drift are not explicitly modeled in the current evaluation. Addressing these challenges will involve incorporating robust federated aggregation, client reputation mechanisms, drift-aware retraining strategies, and life-long learning techniques to mitigate catastrophic forgetting and maintain detection performance over time.

Overall, PrivEdge provides a solid foundation for privacy-preserving, real-time electricity theft detection at the edge. The outlined future directions aim to strengthen its robustness, scalability, and adaptability, supporting its potential for sustained deployment in next-generation smart-grid infrastructures.

## Data Availability

The datasets analyzed during the current study are publicly available: https://github.com/henryRDlab/ElectricityTheftDetection. The code used for model implementation and analysis is not publicly available due to privacy and institutional policy restrictions but can be provided by the corresponding author upon reasonable request.
